# Interaction of the heterotrimeric G protein alpha subunit SSG-1 of *Sporothrix schenckii *with proteins related to stress response and fungal pathogenicity using a yeast two-hybrid assay

**DOI:** 10.1186/1471-2180-10-317

**Published:** 2010-12-09

**Authors:** Lizaida Pérez-Sánchez, Elizabeth González, Emilee E Colón-Lorenzo, Waleska González-Velázquez, Ricardo González-Méndez, Nuri Rodríguez-del Valle

**Affiliations:** 1 Department of Microbiology and Medical Zoology, Medical Sciences Campus, University of Puerto Rico, PO Box 365067, San Juan, PR 00936-5067, USA; 2 Department of Radiological Sciences, Medical Sciences Campus, University of Puerto Rico, PO Box 365067, San Juan, PR 00936-5067, USA

## Abstract

**Background:**

Important biological processes require selective and orderly protein-protein interactions at every level of the signalling cascades. G proteins are a family of heterotrimeric GTPases that effect eukaryotic signal transduction through the coupling of cell surface receptors to cytoplasmic effector proteins. They have been associated with growth and pathogenicity in many fungi through gene knock-out studies. In *Sporothrix schenckii*, a pathogenic, dimorphic fungus, we previously identified a pertussis sensitive G alpha subunit, SSG-1. In this work we inquire into its interactions with other proteins.

**Results:**

Using the yeast two-hybrid technique, we identified protein-protein interactions between SSG-1 and other important cellular proteins. The interactions were corroborated using co-immuneprecipitation. Using these techniques we identified a Fe/Mn superoxide dismutase (SOD), a glyceraldehyde-3-P dehydrogenase (GAPDH) and two ion transport proteins, a siderophore-iron transporter belonging to the Major Facilitator Superfamily (MFS) and a divalent-cation transporter of the Nramp (natural resistance-associated macrophage protein) family as interacting with SSG-1. The cDNA's encoding these proteins were sequenced and bioinformatic macromolecular sequence analyses were used for the correct classification and functional assignment.

**Conclusions:**

This study constitutes the first report of the interaction of a fungal G alpha inhibitory subunit with SOD, GAPDH, and two metal ion transporters. The identification of such important proteins as partners of a G alpha subunit in this fungus suggests possible mechanisms through which this G protein can affect pathogenicity and survival under conditions of environmental stress or inside the human host. The two ion transporters identified in this work are the first to be reported in *S. schenckii *and the first time they are identified as interacting with fungal G protein alpha subunits. The association of G protein alpha subunits to transport molecules reinforces the role of G proteins in the response to environmental signals and also highlights the involvement of fungal G protein alpha subunits in nutrient sensing in *S. schenckii*. These interactions suggest that these permeases could function as transceptors for G proteins in fungi.

## Background

*Sporothrix schenckii *is a human and animal pathogen belonging to the family Ophiostomataceae [1]. While this family of fungi includes important plant pathogens, *S. schenckii *is a human pathogen commonly found in soil or vegetation with infections commonly seen in agricultural workers and gardeners. It is the etiologic agent of a disease known as sporotrichosis, an important cutaneous lymphatic mycosis with a worldwide distribution [2-4]. *S. schenckii *is dimorphic and can grow either in a mycelial form with long branching filaments at 25°C or in the form of spherical ovoid yeast cells which are typically found in animal hosts [1].

In nature or in animal hosts, fungal cells must respond efficiently to changing environmental conditions in order to survive. Cell membrane receptors play an essential role in the response of the fungal cell to the environment. Information is conveyed to the interior of the cell following the binding of ligands to receptors. The heterotrimeric G proteins constitute a family of GTPases that transmit messages received at cell surface receptors (GPCR) to cytoplasmic effector proteins inside the cell [5]. Heterotrimeric G proteins are made up of three subunits: the GTP-binding α subunit and the tightly associated complex of β and γ subunits. Once a ligand binds to a receptor, the heterotrimeric G proteins are activated, initiating the exchange of GDP to GTP in the Gα subunit causing a conformational change that results in the dissociation of the heterotrimer into Gα-GTP and Gβγ subunits. The Gα-GTP and/or Gβγ subunits interact with effector proteins such as enzymes or ion channels, resulting in the regulation of a broad range of cellular processes and pathways [6-10]. Many genes encoding heterotrimeric G protein subunits have been described in fungi. GPA-like G protein α subunits are present in: *Saccharomyces cerevisiae *[11-13], *Cryptococcus neoformans *[14] and *Candida albicans *[15,16], and in the plant pathogens *Ustilago maydis *[17], among others. Gα subunits similar to the traditional Gα class rather than to the GPA group have been described in the filamentous fungi and plant pathogens such as *Aspergillus nidulans *[18], *Neurospora crassa *[19-21], *Cryphonectria parasitica *[22,23], and *Magnaporthe grisea *[24].

In *S. schenckii*, we reported the first member of the Gαi family in a human pathogenic fungus [25]. The cDNA of *ssg-1 *encoded a 353 amino acids pertussis toxin sensitive Gαi subunit of 41 kDa. Subsequently, we identified and sequenced two new G protein alpha subunit genes in this fungus encoding SSG-2 [26] and SSG-3 (mRNA GenBank accession no. AY957584). The *ssg-2 *cDNA encoded a protein with 355 amino acids and a molecular weight of 40.90 kDa. The *ssg-3 *cDNA encoded a protein with 354 amino acids and a predicted molecular weight of 40.87 kDa. These three proteins have the consensus sequences that identify Gα subunits, which are the five highly conserved domains that form the guanine nucleotide binding site that define the Gα protein superfamily [27].

Gα subunits have been implicated in the regulation of fungal development and pathogenicity mostly based on the evidence derived from gene knock-out studies. In *N. crassa*, deletion of the Gαi homologue *gna-1*, results in impaired proliferation, defective macroconidiation, and production of abnormal female reproductive structures. A second Gα subunit gene in *N. crassa*, *gna-2*, has overlapping functions with *gna-1*, as demonstrated by a double deletion assay [20]. The third Gα subunit gene in *N. crassa *is *gna-3*. Mutants of *gna-3 *share several phenotypes with the adenylyl cyclase mutants such as premature conidiation, short aerial hyphae and reduced ascospore viability [21].

Strains of the chestnut blight fungus *C. parasitica*, harboring RNA viruses exhibit reduced levels of virulence, which were attributed to lower levels of the Gαi subunit CPG-1 [22]. Disruption of *cpg-1 *affects hyphal growth, conidiation, female fertility, and virulence. Disruption of a second G protein α subunit gene, *cpg-2*, resulted in a slight reduction of growth rate and asexual sporulation, but no significant reduction in virulence [28]. Further testing of G protein subunits in *C. parasitica *revealed a third Gα homologue, CPG-3, but its functions have not been determined [23].

*M. grisea*, the fungal pathogen that causes rice blast disease, has three Gα subunits [24]. Disruption of the Gαi subunit gene, *magB*, reduces vegetative growth, conidiation, appressorium formation, pathogenicity, and blocks sexual development [29]. Also, the targeted deletion of a regulator of G protein signalling, MoRIC8, which interacts with the pertussis sensitive MagB alpha subunit, rendered the fungus non-pathogenic [30]. Disruption of the two other Gα subunit genes, *magA *and *magC*, affected latter stages of sexual development [24].

In *U. maydis*, which causes corn smut disease, four genes encoding Gα subunits, *gpa1 *to *gpa4*, have been described [17]. The Gpa1, Gpa2, and Gpa3 have homologues in other fungal species, but the Gpa4 is unique to this fungus. Gpa3 is most closely related to the GPA-1 of *C. neoformans *(75% identity), and is required for *U. maydis *pathogenicity, and mating [31].

The studies mentioned above are a few examples of the work done on the role of Gα subunits in the biology of fungi. Specifically they demonstrate a role for these subunits in the response to stressful conditions and pathogenicity. Nevertheless, the actual proteins with which these Gα subunits interact have not been identified. Our initial inquiry into the protein-protein interactions involving heterotrimeric G protein alpha subunits was done using SSG-2 as bait. In this case, we identified a cytoplasmic phospholipase (cPLA_2_) homologue interacting with this Gα subunit [26]. This was the first report of a G protein alpha subunit interacting with a protein directly related to pathogenicity in fungi. PLA_2 _was also found to be necessary for the expression of the dimorphic potential of *S. schenckii *[26].

In this work, we inquired into the proteins interacting with the *S. schenckii *pertussis sensitive G protein alpha subunit, SSG-1, using the yeast two-hybrid assay. We identified proteins related to the response of fungi to stressful conditions and pathogenicity. The identification of such important proteins as partners of SSG-1 offers evidence on how this Gα subunit can affect survival of the fungus in the human or animal host and enhances our knowledge of the mechanisms involved in the disease producing processes of fungi.

## Results

More than 60 inserts from colonies growing in quadruple drop out medium (QDO) (SD/-Ade/-His/-Leu/-Trp/X-α-gal) from two different *S. schenckii *yeast cDNA libraries were analyzed for the presence of SSG-1 interacting proteins. Only inserts from colonies that grew in QDO and were positive for X-α-gal were cloned and sequenced. Four of these colonies were chosen for further characterization because the inserts were identified as encoding proteins related to survival in stressful conditions and/or pathogenicity in many microorganisms, specifically fungi [32-36]. These inserts encoded the C-terminal domains of a mitochondrial superoxide dismutase (SOD), a cation transporter of the Nramp family, a sidereophore-iron transporter and glyceraldehyde-3-P dehydrogenase (GAPDH).

### Genetic and bioinformatic characterization of *S. schenckii *SOD (SsSOD)

The sequence obtained by PCR from the insert in colony number 21 showed a 463 bp product and a derived amino acid sequence of 17 amino acids containing part of an Fe/Mn SOD C-terminal domain. The TAG stop codon at the end of the coding sequence was followed by a 387 bp 3'UTR and a 27 bp poly A^+ ^tail. The online BLAST algorithm [37] matched the sequence to the C-terminal domain of superoxide dismutase from *Aspergillus fumigatus *(GenBank no. EAL88576.1).

**Figure 1:**
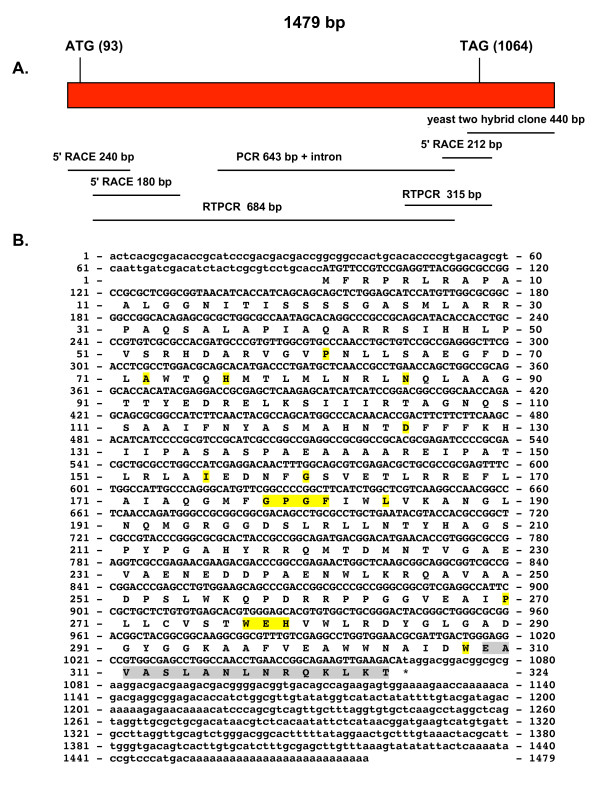
**cDNA and derived amino acid sequences of the *S. schenckii sssod *gene**. Figure 1A shows the sequencing strategy used for the *sssod *gene. The size and location in the gene of the various fragments obtained from PCR and RACE are shown. Figure 1B shows the cDNA and derived amino acid sequence of the *sssod *gene. Non-coding regions are given in lower case letters, coding regions and amino acids are given in upper case letters. The conserved residues are shadowed in yellow. The original sequence isolated using the yeast two-hybrid assay is shadowed in gray.

The sequencing strategy used to complete the coding sequence of the *sssod *cDNA is shown in Figure 1A. The cDNA and coding sequence were completed (GenBank accession numbers: DQ489720 and ABF46644.3) as shown in Figure 1B using 5'RACE. This figure shows a cDNA of 1479 bp with an ORF of 972 bp encoding a 324 amino acid protein with a calculated molecular weight of 35.44 kDa. The PANTHER Classification System [38] identified this protein as a member of the SOD2 family (PTHR11404:SF2) (residues 26-319) with an extremely significant E value of 2.4 e^-66^. Figure 1B does not show the characteristic histidine residues that are part of the metal ion binding site in human SOD2 (GenBank accession no. NP_000627), H26 and H73. In *S. schenckii*, H73 is substituted by D125. Another metal binding residue, present in human SOD2, D159 is absent from this protein and its homologues (Figure 1 and also Additional File 1). In *S. schenckii*, it is substituted by S275 and N in all other fungal homologues (Additional File1). Another metal binding residue, H163 in human SOD2 is present in *S. schenckii *as H279. Residues that are present in 100% of the SODs and the GXGX signature (present as GPGF) are shadowed in yellow in Figure 1B.

A mitochondrial targeting sequence was identified using PSORT II [39], with a putative cleavage site at amino acid 53 (SRH/DA) and a probability of it being mitochondrial of 52.5% *vs*. a probability of it being cytoplasmic of 21.7%. PSORT II [39] also identified an endoplasmic reticulum (ER) membrane modified retention signal at the N-terminus (FRPR) and the C-terminus (QKLK). The TargetP 1.1 Server [40] predicted a shorter mitochondrial signal peptide with a length of 45 amino acids. This signal peptide length is more in accordance with the structure of other members of the SOD2 family.

A multiple sequence alignment of the derived amino acid sequence of SsSOD to other fungal SOD homologues and the human SOD2 is included in Additional File 1. BLAST search for the deduced amino acid sequence identified this protein as approximately 40% identical to a Fe/Mn SODs of fungi such as: *Chaetomium globosum*, *Gibberella zeae *and *M. grisea*, among others (Additional File 2, Supplemental Table S1).

### Genetic and bioinformatic characterization of *S. schenckii *Nramp (SsNramp)

The insert in colony number 156 was identified as the C-terminal domain of an Nramp (Smf1/Smf2) homologue after sequencing. This insert was preliminarily identified as a sequence that matched with Nramp transporters from *A. fumigatus *(GenBank no. XP_751729.2) using the online BLAST algorithm [37].

**Figure 2:**
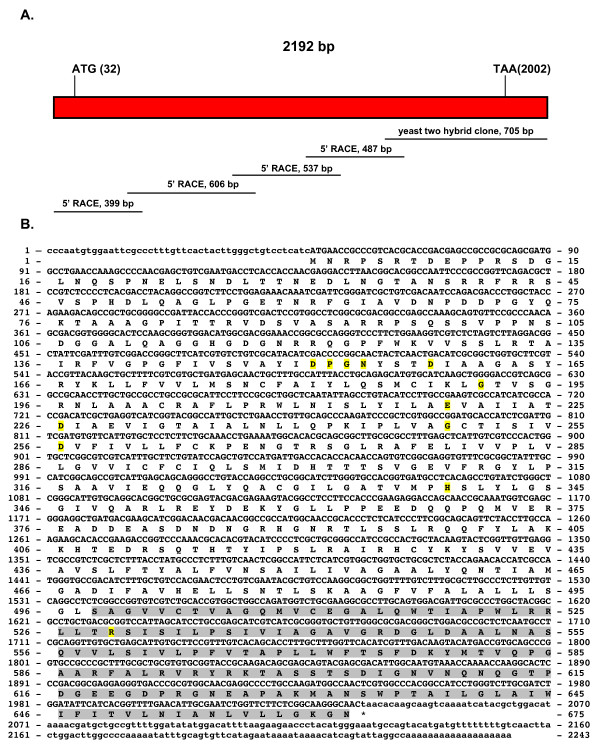
**cDNA and derived amino acid sequences of the *S. schenckii ssnramp *gene**. Figure 2A shows the sequencing strategy used for the *ssnramp *gene. The size and location in the gene of the various fragments obtained from RACE are shown. Figure 2B shows the cDNA and derived amino acid sequence of the *ssnramp *gene. Non-coding regions are given in lower case letters, coding regions and amino acids are given in upper case letters. The conserved residues are shadowed in yellow. The original sequence isolated using the yeast two-hybrid assay is shadowed in gray.

The coding sequence of the *ssnramp *cDNA was completed using 5' RACE as shown in Figure 2A (GenBank accession numbers: GQ411366.1 and ACV31218.1). Figure 2B shows the 2243 bp cDNA with an ORF of 1989 bp encoding a 663 amino acid protein with a calculated molecular weight of 71.41 kDa. This figure also shows the sequence of the original insert isolated from colony156 shadowed in gray that consisted of 498 bp ORF followed by a 185 bp 3'UTR and 19 bp poly A^+ ^tail.

The invariant residues are highlighted in yellow in Figure 2B. These include residues: D151 (86 in mouse Nramp2), E219 (154 in mouse Nramp2), H339 (267 in mouse Nramp2) and R524 (416 in mouse Nramp2), and the highly conserved residues: D226 (161 in mouse Nramp2) and D256 (192 in mouse Nramp2). G191 is also conserved in all Nramp homologues and in SsNramp it corresponds to G249. The amino acid sequence, DPGN, constitutes an Nramp invariant motif and is present in SsNramp (amino acids 151-154) and its homologues. This motif is located between TM helix 1 and TM helix 2 and is extra-cytoplasmic as expected.

**Figure 3:**
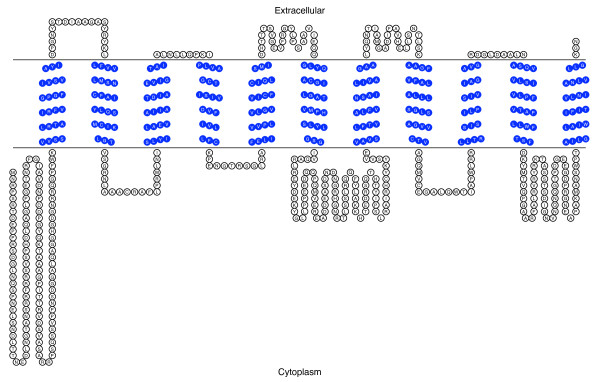
**Transmembrane domain analysis of SsNramp**. Figure 3 shows the transmembrane domain analysis of SsNramp. This figure shows the 11 predicted transmembrane helices in SsNramp that characterize this transporter family. Predictions were made with TMHMM and results were visualized with TOPO2.

Using the PANTHER Classification System [38] to analyze the deduced amino acid sequence, we identified this protein as a metal transporter of the Nramp family (PTHR11706:SF11) with an E value of 1.5 e^-245^. Blocks server analysis showed natural resistance-associated macrophage protein signature from amino acids 214 to 575. PSORT II analysis [39] of this Nramp homologue suggests that it resides in the plasma membrane with 65.2%, plasma membrane *vs*. 30.4% endoplasmic reticulum. Using the TMHMM Server we found the 11 transmembrane helices that characterize this transporter family as shown in Figure 3.

A multiple sequence alignment of the derived amino acid sequence SsNramp and other fungal homologues is included as Additional File 3. The percent identity of SsNramp to that of other fungi such *N. crassa*, *S. cerevisiae *and *Coccidioides posadasii *among others, is in the range of 47 to 56% (Additional File 2, Supplemental Table S2).

### Genetic and bioinformatic characterization of *S. schenckii *Sit (SsSit)

The online BLAST algorithm matched the sequence obtained from the insert in colony number 435 with a putative siderophore transporter from *A. fumigatus *(GenBank accession number EAL86419.1) [37]. This insert contained 370 bp and encoded 98 amino acids of a siderophore-iron transporter C-terminal domain followed by a 45 bp 3'UTR.

**Figure 4:**
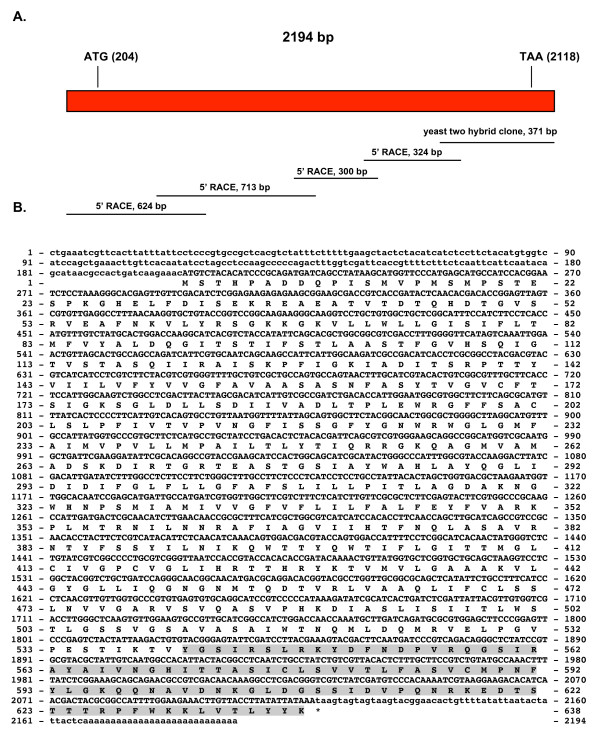
**cDNA and derived amino acid sequences of the *S. schenckii sssit *gene**. Figure 4A shows the sequencing strategy used for *sssit *gene. The size and location in the gene of the various fragments obtained from PCR and RACE are shown. Figure 4B shows the cDNA and derived amino acid sequence of the *sssit *gene. Non-coding regions are given in lower case letters, coding regions and amino acids are given in upper case letters. The original sequence isolated using the yeast two-hybrid assay is shadowed in gray.

The sequencing strategy used for obtaining the cDNA coding sequence of the *sssit *gene homologue was based on 5'RACE, shown in Figure 4A. This figure shows a cDNA of 2194 bp with an ORF of 1914 bp encoding a 638 amino acid protein with a calculated molecular weight of 69.71 kDa (GenBank accession numbers: GQ411365 and ACV31217). The PANTHER Classification System [38] identified this protein as a siderophore-iron transporter 3 of the Major Facilitator Superfamily (PTHR24003:SF129) (residues 109-529) with an extremely significant

**Figure 5:**
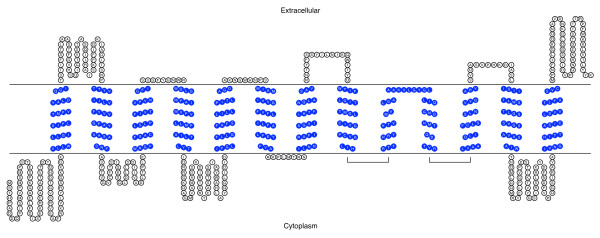
**Transmembrane analysis of the *S. schenckii *siderophore-iron transporter**. Figure 5 shows the transmembrane domain analysis of SsSit. Thirteen transmembrane helices were predicted using TMHMM. TMHMM results were visualized with TOPO2.

E value of 2.1e^-78 ^[38]. Using the TMHMM Server we found 13 transmembrane helices as shown in Figure 5. The number and localization of the transmembrane helices fluctuated between 11 and 13 helices, depending on the transmembrane helix prediction server used. Further studies will be needed to address these discrepancies, therefore, the predicted membrane topology must be considered to be speculative. All prediction servers coincide in the identification of the 11 TM helices shown in Additional File 4 containing the multiple sequence alignment. PSORT II analysis [39] classifies this transporter as residing in the plasma membrane (78.3%: plasma membrane *vs*. 21.7%: endoplasmic reticulum).

In Additional File 4, multiple sequence alignment of the derived amino acid sequence *sssit *and other siderophore-iron transporter homologues from fungi such as *G. zeae*, *C. globosum *and *Aspergillus flavus *is shown. The percent identity of SsSit varied considerably between the *S. schenckii *transporter and that of other fungi. The highest percent identity was approximately 74% to that of *G. zeae *(Additional File 2, Supplemental Table S3).

### Genetic and bioinformatic characterization of *S. schenckii *GAPDH (SsGAPDH)

A GAPDH homologue identified as being present in the surface of various fungi, was the insert from colony number 159 [36]. This insert had 697 bp and encoded a140 amino acid sequence. This represented almost half of the amino acid sequence of GAPDH and a 274 bp 3'UTR. The online BLAST algorithm matched the sequence with GAPDH from *G. zeae *(GenBank acession number XP_386433.1) with 87% identity in the C-terminal region [37].

**Figure 6:**
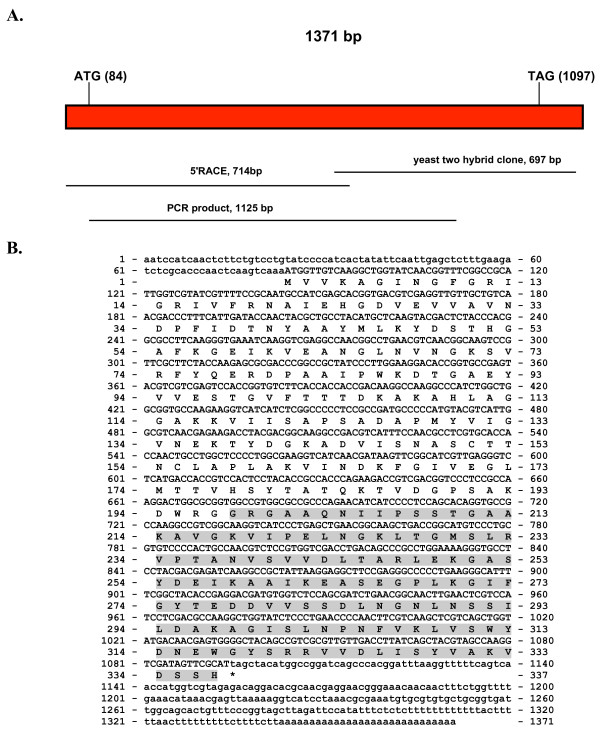
**cDNA and derived amino acid sequences of the *S. schenckii ssgapdh *gene**. Figure 6A shows the sequencing strategy used for *ssgapdh *gene. The size and location in the gene of the various fragments obtained from PCR and RACE are shown. Figure 6B shows the cDNA and derived amino acid sequence of the *ssgapdh *gene. Non-coding regions are given in lower case letters, coding regions and amino acids are given in upper case letters. The original sequence isolated using the yeast two-hybrid assay is shadowed in gray.

Figure 6A shows the sequencing strategy used for obtaining the cDNA coding sequence of the *gapdh *gene homologue. Figure 6B shows a cDNA of 1371 bp with an ORF of 1011 bp encoding a 337 amino acid protein with a calculated molecular weight of 35.89 kDa (GenBank accession numbers: GU067677.1 and ACY38586.1). The PANTHER Classification System [38] identified this protein as glyceraldehyde-3-P-dehydrogenase (PTHR 10836) (residues 1-336) with an extremely significant E value of 3 e^-263^. Pfam [41] identified an NAD binding domain from amino acid 3 to 151 (E value of 5e^-59^) and a glyceraldehyde-3-P dehydrogenase C-terminal domain from amino acid 156-313 (E value of 3.1e^-74^). Prosite Scan search identified a GAPDH active site from amino acids 149 to 156 [42,43].

A multiple sequence alignment of SsGAPDH to other GAPDH fungal homologues such as those from *M. grisea*, *G. zeae *and *C. globosum *is given in Additional File 5. This figure shows the extremely high degree of conservation among these proteins in the range of 71 to 87% (Additional File 2, Supplemental Table S4).

### Confirmation of the SSG-1-protein interactions by co-immunoprecipitation and Western blot

**Figure 7:**
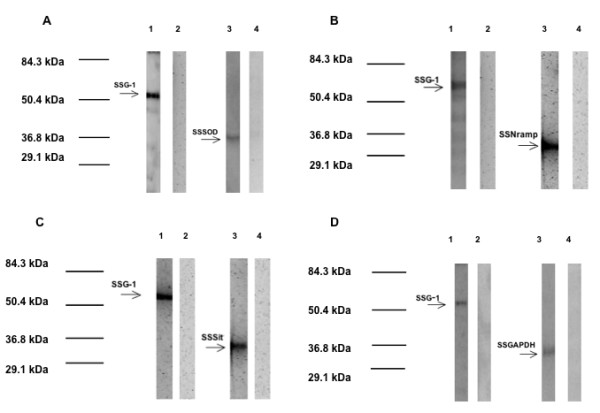
**Co-immunoprecipitation and Western Blot analyses of SSG-1 interacting proteins**. Whole cell free extracts of *S. cerevisiae *cells expressing the complete c-myc tagged SSG-1 coding sequence fused to the GAL4 activation domain (bait protein) and the HA tagged protein fragment fused to the GAL4 DNA binding domain (prey protein) were co-immunoprecipitated as described in Methods. The co-immuneprecipitated proteins were separated using 10% SDS polyacrylamide electrophoresis and transferred to nitrocellulose. The nitrocellulose strips were probed with anti-cMyc antibodies (Lane 1) and anti HA antibodies (Lane 3). Pre-stained molecular weight markers were included in outside lanes of the gel. The position of the molecular weight markers is indicated in the figure. Lanes 2 and 4 are negative controls where no primary antibody was added. Figure 7A corresponds to the results of the Co-IP of SSG-1 and SsSOD, Figure 7B corresponds to the results of the Co-IP of SSG-1 and SsNramp, Figure 7C corresponds to the results of the Co-IP of SSG-1 and SsSit and Figure 7D corresponds to the results of the Co-IP of SSG-1 and SsGAPDH.

Figure 7 shows the confirmation of the protein-protein interactions by using co-immunoprecipitation (Co-IP) and Western blots. The results of independent Co-IPs for each of the different SSG-1 interacting proteins are shown. In all co-immunoprecipitation and Western blot analyses, SSG-1 was observed as a band with a calculated molecular weight of 59.8 ± 1.5 kDa, always within less than 1 standard deviation of the average. The calculated theoretical value, considering that SSG-1 was expressed fused to the GAL-4 binding domain, was 61.1 kDa. In all graphics shown in Figure 7, lanes 2 and 4 present the negative controls as described herein. Lane 2 shows the results obtained in the Western blot when the primary anti-cMyc antibody was not added (negative control). Lane 4 shows the results obtained in the Western blot when the primary anti-HA antibody was not added (negative control).

Figure 7A shows the confirmation of the interaction observed in the yeast two-hybrid assay between SSG-1 and SsSOD by Co-IP and Western blot analysis. Lane 1 shows the band obtained using anti-cMyc antibody that recognizes SSG-1. Lane 3 shows the band obtained using anti-HA antibody that recognizes the SsSOD fragment (amino acids 260 to 324). The observed molecular weight of this band is 33.5 kDa and is slightly higher than the theoretical value (26.5 kDa), calculated considering that only the last 65 amino acids of the protein were present and that this fragment was fused to the GAL-4 activation domain (Additional File 2, Supplemental Table S5). This difference between the observed and the theoretical molecular weight could be due to sodium dodecyl sulfate (SDS) binding because of the large number of hydrophobic and basic residues in this protein fragment. It could also be the effect of post-translational modifications of the peptide which might include myristoylation and phosphorylation (Prosite Scan analysis) [42-44].

The results that confirm the interaction observed between SSG-1 and SsNramp by Co-IP and Western blot analysis are shown in Figure 7B. Lane 1 shows the band obtained using anti-cMyc antibody that identified SSG-1. Lane 3 shows the band obtained using anti-HA antibody that recognizes the original SsNramp C-terminal domain isolated from the yeast two-hybrid clone. This band is of the expected size (35.5 kDa) because the original insert contained the last 165 amino acids of the protein fused to the GAL-4 activation domain (Additional File 2, Supplemental Table S5).

Co-immunoprecipitation and Western blot analysis shown in Figure 7C confirmed the interaction observed in the yeast two-hybrid assay between SSG-1 and SsSit. Lane 1 shows the band obtained using anti-cMyc antibody that recognizes SSG-1. Lane 3 shows the band obtained using anti-HA antibody that recognizes the original SsSit fragment isolated from the yeast two-hybrid clone. This band is of the expected size (33.2 kDa) taking into consideration the molecular weight of the last 177 amino acids of the protein and that of the GAL-4 activation domain (Additional File 2, Supplemental Table S5).

The interaction between SSG-1 and SsGAPDH by co-immunoprecipitation and Western blot analysis is shown in Figure 7D. Lane 1 shows the band obtained using anti-cMyc antibody that recognizes SSG-1. Lane 3 shows the band obtained using anti-HA antibody that recognizes the original SsGAPDH fragment isolated from the yeast two-hybrid clone. This band is of the expected size (35.5 kDa) considering that the insert encoded only the last 140 amino acids of the protein and that the fragment was fused to the GAL-4 activation domain (Additional File 2, Supplemental Table S5).

## Discussion

Heterotrimeric G proteins are universal recipients of environmental signals in all living eukaryotic cells [45]. Genes encoding G protein subunits have been extensively studied in fungi [46], but in there is limited information available regarding heterotrimeric G proteins signalling pathways in the pathogenic fungi other than that related to the cAMP dependent pathway. Further inquiry is needed to comprehend the full scope of G protein signalling pathways in pathogenic fungi. An important way to discover other signalling pathways involving heterotrimeric G proteins is to study protein-protein interaction. This study was aimed at identifying important components of the G protein alpha subunit SSG-1 signalling using a yeast two-hybrid screening approach. More than 30 potential interacting proteins were identified but we chose to corroborate and inform the interactions of *S. schenckii *homologues of four very important proteins: SOD, Nramp, Sit1 and GAPDH. All of these proteins have been identified in other fungi as being involved in pathogenicity or environmental stress response as will be discussed below.

The superoxide dismutase (SOD) identified as interacting with SSG-1 belongs to a family of enzymes that catalyze the dismutation of oxygen radical to hydrogen peroxide eliminating superoxide anions generated in aerobic respiration [47,48]. Many SOD genes have been identified in fungal genomes [49]. SODs have been shown to contribute to growth and survival of fungi under oxidative stress conditions, specifically inside macrophages. In *C. neoformans*, SOD1 mutants were observed to be less virulent while SOD2 mutants had increased susceptibility to oxidative stress and showed decreased growth at elevated temperatures [50,51]. Virulence in *C. neoformans *variety *gattii *has been reported to be dependent on both SOD1 and SOD2 [32,33]. In *C. albicans *the null mutant of mitochondrial SOD2 was more sensitive than wild-type cells to stress [52] and the SOD1 null mutant had attenuated virulence [53].

*S. schenckii *superoxide dismutases have not been studied. In fact, this is the first report of the presence of a member of this protein family in this fungus. Analysis of the amino acid sequence of SsSOD against the *Homo sapiens *database using BLAST shows that it is homologous to the human manganese superoxide dismutase SOD2 family with 32% identity. This same analysis, using the fungal databases revealed that SsSOD is phylogenetically closely related to SODs of the filamentous fungi with the sequence identity being in the range of 23-43%. Also SsSOD has a calculated molecular weight of 35.44 kDa, very close to that of other fungal homologues. The specific role of SOD2 in *S. schenckii *stress and pathogenesis has yet to be addressed.

Fungal SODs have two main locations: cytosolic or mitochondrial [49]. Analysis using PSORT II [39] and TargetP [40] suggests that SsSOD isolated by the yeast two-hybrid analysis is a mitochondrial SOD. Being a mitochondrial protein does not disqualify SsSOD as an interacting partner of SSG-1. It is important to note that Gαi subunits can be present not only in the cytoplasm but also in the mitochondria [54]. Also, SODs acquire the metal ion during protein synthesis and this seems to occur in the cytoplasmic face of the mitochondrial membrane. It is also of interest to note that another mitochondrial protein was also found to interact with SSG-1 (unpublished results). This protein belongs of the mitochondrial metal transporter protein family (Mtm family) that is known to be involved in the acquisition of the metal ion by SODs [55,56]. These results together with the interactions of SSG-1 and the metal ion transporters SsNramp and SsSit, discussed below suggest a possible role of SSG-1 in SODs metal acquisition.

Metals are essential nutrients and important co-factors of a variety of proteins and enzymes; they are required for the survival of all organisms. Fungi have developed multiple strategies to acquire metals from the environment [57]. The human host is a hostile environment for invading pathogens because it actively sequesters and limits nutrients [58]. The term nutritional immunity has been coined to describe metal ion sequestration [59]. In this work we have identified a homologue of the Nramp family of cation transporters present in higher organisms and yeasts [60,61] as interacting with SSG-1. This family of transporters is associated with virulence in bacteria and to resistance to infection in mammalian hosts [34,62]. The Nramp family specifically transports manganese and iron although they have the capacity to transport other divalent cations such as nickel, zinc, copper, cobalt and cadmium [60]. They are characterized by a hydrophobic core with 10-12 transmembrane helices [61], also present in the *S. schenckii *homologue described here. The Nramp family consists of Nramp1, Nramp2, and the yeast proteins Smf1, Smf2 and Smf3 [60,63]. Smf1 and Smf2 are believed to be involved in manganese homeostasis. Smf1 is a cell surface manganese transporter [56,63]. The *S. schenckii *Nramp described here is more closely related to Smf1, it is similar in size to Smf1 and is predicted to be located in the plasma membrane by PSORT II analysis [39]. Although there is considerable similarity between SsNramp and Smf1, SsNramp's role in cation transport must be elucidated and its substrate identified.

Another critical aspect for the survival of fungal pathogens inside the host is the capacity to accumulate iron [64]. In this work we report a siderophore-iron transporter as interacting with SSG-1. In response to low iron availability, most fungi synthesize siderophores that chelate iron which is ultimately taken up as a siderophore-iron complex [65,66] by members of the Major Facilitator Superfamily transporters (MSF) [65,67]. Members of the MFS do not possess well-defined conserved motifs as it is known from other transporter superfamilies but the Panther Classification System identified SsSit1 as a siderophore iron transporter. Studies in *C. albicans *revealed a role for a siderophore iron transporter (SIT1) in epithelial invasion. Gene knock-out studies of *sit1 *led to a reduction in the invasion and penetration of epithelia by this fungus [35]. In *C. neoformans*, SIT1 has a role in the structure of the cell wall and melanization [68].

It is of interest to note that *S. schenckii *is capable of producing its own siderophores, unlike *S. cerevisiae *that does not [66,69]. The identification of the relationship between siderophore iron transport and a Gα subunit opens a new angle to the already complex regulation of iron uptake in fungi and identifies G proteins as potentially important players in the tightly regulated mechanism of iron acquisition.

The reported interaction of these two ion transport proteins with SSG-1 in *S. schenckii *is a key factor discussed here. In addition to the ion transporters reported in this work, SSG-1 has been observed to interact with a monosaccharide transporter of the MFS family (unpublished results) and SSG-2 can interact with a hexose transporter of this same family of proteins (unpublished results). It is a known fact that heterotrimeric G proteins interact with classical receptor proteins in the membrane resulting in the activation of signal transduction pathways. However, it has been observed that nutrient carriers can also function as receptors for signalling [70,71]. The activation of signal transduction pathways by nutrients has been recognized in other systems mainly, *S. cerevisiae *[72]. Yet, many of the primary intracellular receptors of the signals generated through nutrient carriers have not been identified. In this paper we offer evidence that links transport molecules to G protein signalling and suggests that G proteins could be one of the effectors of nutrient sensing in fungi. There is a hypothesis that GPCR receptors may have evolved from nutrient transporters that gradually lost their transport capacity [71]. Our findings provide a new avenue to study this evolutionary hypothesis.

Another SSG-1 interacting protein identified in this work was GAPDH, a highly conserved fungal protein as shown in Additional File 5. The presence of GAPDH, a glycolytic enzyme, on the surface of fungal cells has been reported for various fungal species, such as *C. albicans *[73] and *Paracoccidiodes braziliensis *[36]. This alternative localization of the enzyme suggests other roles for this protein besides glycolysis, possibly related to pathogenesis and stress response. In *P. braziliensis*, this enzyme has been identified as important in the adhesion to pneumocytes [36] while in *S. cerevisiae*, GAPDH was reported to affect survival under condition of oxidative stress as a target for S-thiolation, [74]. In *Schizosaccharomyces pombe *GAPDH was transiently oxidized in response to hydrogen peroxide, enhancing the association between a response regulator and MAPKKK's promoting peroxide stress signalling [75]. The association of GAPDH to SSG-1 offers additional information to be considered when assessing the role of GAPDH outside of its traditional function as a glycolytic enzyme.

The actual identification of protein-protein interactions constitutes a very important and necessary step if we are to understand the role of G proteins in fungal signalling pathways. The results presented in this paper suggests the involvement of SSG-1 with proteins whose role in many other fungi have been recognized as part of the protective mechanism against the strain that both the environment and the human host pose for the survival of the fungus.

## Conclusions

This study constitutes the first report of the protein-protein interactions of the fungal Gαi subunit SSG-1 with cellular proteins. SOD, GAPDH, and two metal ion transporters were identified as SSG-1 interacting proteins and these interactions were confirmed using Co-IP. The identification of such important proteins as partners of a Gα subunit in this fungus suggests possible mechanisms through which this G protein can affect pathogenesis or survival under conditions of stress and nutrient limitation inside the human host or the environment. These proteins belong to different families and have different but well-established roles, yet all converge in a common role: involvement in the response to stress. Individually, SOD2 is well known as a major player in the elimination of ROS in all cells while GAPDH has been recognized as promoting resistance to oxidative stress in fungi. The two ion transporters identified in this work are important in overcoming the metal ion limitations imposed on invading pathogens by the human or animal host as a defence mechanism and provide the necessary metal co-factors for SODs and other important proteins. The association of G protein alpha subunits to transport molecules reinforces the role of G proteins in the response to environmental signals and also highlights the involvement of fungal G protein alpha subunits in nutrient sensing in *S. schenckii*. These interactions suggest that these permeases could function as transceptors for G proteins in fungi.

## Methods

### Strains and culture conditions

*S. schenckii *(ATCC 58251) was used for all experiments. The yeast form of the fungus was obtained from conidia as previously described [76]. *S. cerevisiae *strains AH109 and Y187 were used for the yeast two-hybrid screening and were supplied with the MATCHMAKER Two-Hybrid System (Clontech Laboratories Inc., Palo Alto, CA, USA).

### Nucleic acids isolation

Total RNA was obtained from *S. schenckii *yeast cells as described previously by us [25]. Poly A^+ ^RNA was obtained from total RNA using the mRNA Purification Kit from Amersham Biosciences (Piscataway, NJ, USA).

### Yeast two-hybrid assay

MATCHMAKER Two-Hybrid System was used for the yeast two-hybrid assay using all 3 different reporter genes for the confirmation of truly interacting proteins (Clontech Laboratories Inc.). For the construction of the SSG-1 bait plasmid, a pCR^®^2.1-TOPO^® ^plasmid (Invitrogen Corp. Carlsbad, CA, USA) containing the *ssg-1 *gene cDNA sequence of *S. schenckii *from the laboratory collection was used as template for PCR to obtain the coding sequence of the *ssg-1 *gene. *E. coli *TOP10F' One Shot^® ^chemically competent cells (Invitrogen Corp.) containing the plasmid were grown in 3 ml of LB broth with kanamycin (50 μg/ml) at 37°C for 12 to 16 hours and the plasmid isolated with the Fast Plasmid™ Mini kit (Brinkmann Instruments, Inc. Westbury, NY, USA). The *ssg-1 *insert was amplified by PCR using primers containing the gene sequence and an additional sequence containing an added restriction enzyme site. The Ready-to-Go™ Beads (Amersham Biosciences, GE Healthcare, Piscataway, NJ, USA) were used for PCR. The forward PCR primer included the adapter sequence added at the 5' end containing the restriction site for Nde I was used to amplify the *ssg-1 *cDNA. The primers used were: SSG-1/NdeI/(fw) 5' ccatatggccatgggttgcggaatgagtgtggaggag 3' and SSG-1 (rev) 5' gataagaccacatagacgcaagt 3'. The *ssg-1 *cDNA sequence with the added restriction enzyme site was cloned again in the same vector, amplified and purified using the QIAfilter Plasmid Purification kit (Qiagen Corp., Valencia, CA, USA). The *ssg-1 *gene was excised from the vector by sequential enzymatic digestion with Nde I and EcoR I. The pGBKT7 plasmid vector was linearized using the same enzymes mentioned above. The restriction digested *ssg-1 *gene and the linearized pGBKT7 were ligated using the Quick Ligation™ Kit (New England Biolabs, Inc., Ipswich, MA, USA). The ligation reaction was centrifuged briefly and incubated at 25°C for 5 min, chilled on ice, and used to transform *E. coli *TOP10F' One Shot^® ^chemically competent cells. The correct orientation and frame of the inserted gene sequence was verified by sequencing analysis. The bait containing plasmid was isolated using Fast Plasmid™ Mini technology (Brinkmann Instruments) and used to transform competent *S. cerevisiae *yeast cells (Y187) with the YEAST-MAKER™ Yeast Transformation System 2 (BD Biosciences, Clontech Laboratories Inc.). Tests for autonomous gene activation and cell toxicity were carried out as described by the manufacturer.

A cDNA library using *S. schenckii *yeast RNA was constructed as described previously in AH109 cells [26]. Transformants were selected in SD/-Leu plates, harvested and used for mating with the bait containing *S. cerevisiae *strain Y187. Mating of *S. cerevisiae *yeast cells strains Y187 (Mat-α) and AH109 (Mat-a) was done according to the manufacturer's instructions as described previously. Colonies growing in triple drop out medium (TDO) (SD/-Ade/-Leu/-Trp) were tested for growth and α-galactosidase production in quadruplet drop out medium (QDO), SD/-Ade/-His/-Leu/-Trp/X-α-gal. Re-plating of these positive colonies into QDO medium was done to verify that they maintain the correct phenotype.

Colony PCR was used to corroborate the presence of both plasmids in the diploid cells using the T7/3'BD sequencing primer pair for the pGBKT7/ssg*-1 *plasmid and the T7/3'AD primer pair for the pGADT7-Rec library plasmid and yeast colony suspension as template. The Ready-to-Go™ Beads (Amersham Biosciences) were used for PCR. The amplification parameters were those described previously [26]. PCR products were analyzed on agarose gels and the DNA recovered using Spin-X Centrifuge Tube Filters as described by the manufacturer (0.22 μm, Corning Costar Corp., Corning, NJ, USA). The PCR products were cloned and amplified as described previously [26]. Plasmid preparations were obtained using the Fast Plasmid™ Mini technology (Brinkmann Instruments) and the inserts sequenced using commercial sequencing services from SeqWright (Fisher Scientific, Houston, TX, USA) and Retrogen (San Diego, CA, USA).

### Co-immunoprecipitation (Co-IP) and Western blots

For Co-IP of SsSOD and SsGAPDH, the C-terminal domains of these proteins previously identified as interacting with SSG-1 in the yeast two-hybrid assay were amplified using cDNA as template and the following primers: SOD-Nde1 (fw) 5' catatgcgcccgccgggcggcgtt and SOD-Xma1 (rev) 5' cccgggtcctatgtcttcaacttc 3' and GAPDH-Nde1(fw) 5' catatggactggcgcggtggccgt 3' and GAPDH-XMA1 (rev) 5' cccgggtgctaatgcgaactatcg 3'. These primers included restriction enzyme sites that enabled the cloning of these fragments into pGADT7AD. Competent yeast cells AH109 were transformed with the cloned fragments and used for mating with Y187 containing plasmid pGBKT7 with the SSG-1 coding insert using the small scale mating protocol as described by the manufacturer. After mating the cells were plated in TDO and them transferred to QDO with X-α-gal. All colonies that grew in QDO and were blue were tested for the presence of both plasmids and the SsSOD and SsGAPDH inserts were sequenced for corroboration of the sequence and correct insertion. For all other Co-IP's the original yeast two-hybrid clones were grown in QDO.

Co-Ip and Western blots were used to confirm the interaction of proteins identified in the yeast two-hybrid analysis with SSG-1 as described previously [26]. *S. cerevisiae *diploids obtained in the yeast two hybrid assay were grown in QDO, harvested by centrifugation and resuspended in 8 ml containing phosphate buffer saline (800 μl) with phosphatase (400 μl), deacetylase (80 μl) and protease inhibitors (50 μl), and PMSF (50 μl). The cells were broken as described previously [77]. The cell extract was centrifuged and the supernatant used for Co-IP using the Immunoprecipitation Starter Pack (GE Healthcare, Bio-Sciences AB, Bjorkgatan, Sweden). Briefly, 500 μl of the cell extract were combined with 1-5 μg of the anti-cMyc antibody (Clontech, Corp.) and incubated at 4°C for 4 h, followed by the addition of protein G beads and incubated at 4°C overnight in a rotary shaker. The suspension was centrifuged and the supernatant discarded, 500 μl of the wash buffer added followed by re-centrifugation. This was repeated 4 times. The pellet was resuspended in Laemmeli buffer (20 μl) and heated for 5 min at 95°C, centrifuged and the supernatant used for 10% SDS PAGE at 110 V/1 h.

Electrophoretically separated proteins were transferred to nitrocellulose membranes using the BioRad Trans Blot System^® ^for 1 h at 20 volts and blocked with 3% gelatin in TTBS (20 mM Tris, 500 mM NaCl, 0.05% Tween-20, pH 7.5) at room temperature for 30-60 min. The strips were washed for with TTBS and incubated overnight in the antibody solution containing 20 μg of antibody, anti-cMyc or anti-HA (Clontech, Corp.). The bait protein (SSG-1) is expressed with a c-myc epitope tag and is recognized by the anti c-myc antibody. The prey proteins are all expressed with an HA epitope tag that is recognized by the anti HA antibody. Controls where the primary antibody was not added were included. The antigen-antibody reaction was detected using the Immun-Star™ AP chemiluminescent protein detection system from BioRad Corporation (Hercules, CA, USA) as described by the manufacturer.

### Sequencing the *sssod*, *ssnramp ssgapdh*, and *sssit *genes

#### Polymerase chain reaction (PCR), Rapid amplification of cDNA ends (RACE) and Reverse transcription Polymerase chain reaction (RTPCR)

The 5' ends of the *S. schenckii sssod*, *ssnramp*, *sssit *and ss*gapdh *gene homologues were obtained using RLM-RACE (Applied Biosystems, Foster City, CA, USA) with *S. schenckii *cDNA as template. All RACE reactions were carried out in the ABI PCR System 2720 (Applied Biosystems). The touchdown PCR and nested PCR parameters used for the initial RACE reactions were the same as described previously [26]. Nested primers were designed to improve the original amplification reactions. Bands from the 5' nested PCR were excised from the gel and cloned as described above. Primers for RACE were designed based on the sequence obtained from the yeast two-hybrid assay. For the initial 5' RACE of *sssod *gene the following primers were used: GSP-UTR-1(rev) 5' actcttctggctgtcaccgtccccgtc 3'; NGSP-UTR-2 (rev) 5' cgccgtccgtcctatgtcttcaacttc 3'; GSP-AWTQHMTLNL (rev) 5' ggttgagcatcagggtcatgtgctgcgtccaggc 3'; NGSP-RSIHHLPV (rev) 5' gacacgggcaggtggtgtatgctgcgg 3'; GSP-HNTDFFFKH (rev) 5' tgcttgaagaagaagtcggtgttgtgg 3' and NGSP-TTYEDREL (rev)

5' ctcttgagctcgcggtcctcgtatgtggtgc 3'. For PCR the primers used were: forward primer WTQYMTL (fw) 5' ttggacccagtacatgaccctgat 3' (obtained from the published sequence of the *G. zeae sod *gene, GenBank accession no. XP_387245.1) and lower primer HVWLRDYG (rev) 5' agcccgtagtcccgcagccacacgtg 3'. For RTPCR the following primers were used: MFRPR (fw) 5' gcaccatgttccgtccgagg 3' and PSLWKQP (rev) 5' ctgcttccacaggctcgggt 3'.

For 5' RACE of *ssnramp *gene the following primers were used: GSP-TASSTSTSDI (rev) 5' ccaatgtcgctcgtactgctcgctgtc 3'; NGSP-TSFDKYMT (rev) 5' cggtcatgtacttgtcaaacgatgtga 3'; NGSP-VVEVAVSLF (rev) 5' aaagagcgagacggcgacctcaacaac 3'; GSP/NGSP-LSMIDHTT (rev) 5' tgtggtgtggtcaatcatggacagc 3' and NGSP-WKVVSSLR (rev) 5' cctaagactagagacgaccttccag 3'. The complete cDNA coding sequence of *ssnramp *was confirmed using RTPCR with cDNA as template and the following primers: UP-1(fw) 5' tgttcactacttgggctgt 3' and LW-1 (rev) 5' gcttgtgttagttgcccttg 3'.

For 5' RACE of the *sssit *gene, the following primers were used: GSP-SVVTLFASV (rev) 5' gacggaagcaaagagtgtaacgacaga 3'; NGSP-SLRKYDFND (rev) 5' tcattgaagtcgtactttcgtaaggat 3'; GSP/NGSP-QLIFCLSS (rev)

5' gggatgaaaggcagaatatgagctgcg 3'; GSP/NGSP-LIHRTTHR (rev)

5' tcggtgtgtggtacggtggattaac 3'; GSP-LEWRGFFS (rev)

5' cgctgaagaagccacgccattccaatg 3'; GSP-TESPKGHE (rev) 5' ctcgtgccctttaggagattccgt 3' and NGSP-STHPAD (rev) 5' gatcatctgcgggatgtgtagaca 3'. The complete cDNA coding sequence of the *sssit *gene was confirmed using RTPCR. cDNA was used as template for RTPCR and the following primers: UP-Sit (fw) 5' ttcaatacagcataacgccactgatc 3' and LW-Sit (rev) 5' aaaacagtgttccgtacttactacta 3'.

For the initial 5' RACE of the *ssgapdh *gene the following primers were used: GAPDH-GMSLRVPTA (rev) 5' gcagtggggacacgcagggacatgccg 3'; NGSP-GAPDH-QNIIPSSTG (rev) 5' ctgtgctggaggggatgatgttctggg 3'. For RTPCR the following primers were used: GPDH-UP-KMVV (fw) 5' caaaatggttgtcaaggc 3' and GAPDH-LW-ISPRI (rev) 5' aaatccgtgggctgatcc 3'.

### Bioinformatics Sequence Analysis

The theoretical molecular weights of the proteins were calculated using the on-line ExPASy tool (http://expasy.org/tools/pi_tool.html). On-line Prosite Scan (Proscan) (http://expasy.org/tools/scanprosite/), Pfam (http://pfam.sanger.ac.uk/search) and Blocks (http://blocks.fhcrc.org/blocks/blocks_search.html) searches were used to identify potential motifs present in SsSOD, SsGAPDH, SsSit and SsNramp [41,43,78]. The protein classification was performed using the PANTHER Gene and Protein Classification System (http://www.PANTHERdb.org) [38]. On-line database searches and comparisons for SsSOD, SsGAPDH, SsSit and SsNramp were performed with Integrated Protein Classification (iProClass) database (http://pir.georgetown.edu/pirwww/dbinfo/iproclass.shtml) [79] and the BLAST algorithm (http://www.ncbi.nlm.nih.gov/BLAST/) with a cutoff of 10^-7^, a low complexity filter and the BLOSUM 62 matrix [37]. Transmembrane helices were identified using the TMHMM Server v. 2.0 (http://www.cbs.dtu.dk/services/TMHMM) [80] and visualized with TOPO2 (http://www.sacs.ucsf.edu/TOPO2/). Cellular localization of the SsSOD and SsNramp was done using the PSORT II Server (http://PSORT.ims.u-tokyo.ac.jp/) [39] and the TargetP 1.1 server (http://www.cbs.dtu.dk/services/TargetP) [40]. Multiple sequence alignments were built using MCOFFEE (http://www.tcoffee.org) [81,82]. The alignments in Additional Files 1 and 3 to 5 were visualized using the program GeneDoc (http://www.psc.edu/biomed/genedoc).

## Authors' contributions

LPS and ECL did the yeast two-hybrid assays that identified SsNramp, SsGAPDH and SsSit as proteins interacting with SSG-1. LPS completed the SsGAPDH, SsNramp and SsSit sequences obtained in the yeast two-hybrid assay, did the co-immunoprecipitation experiments and participated in the bioinformatic study of the proteins. EG cloned SSG-1 in the yeast two-hybrid vector and identified SOD as a SSG-1 interacting protein. WGV constructed the yeast cDNA library for the identification of the Nramp, Sit and GAPDH homologues and contributed to the co-immunoprecipitation studies. RGM participated and supervised the bioinformatic study of the proteins. NRV designed the study, drafted the manuscript, participated in sequence alignments and domain characterization. All authors have read and approved the final manuscript.

## Supplementary Material

Additional file 1 **Protein multiple sequence alignment of SsSOD to other fungal SOD homologues**. Multiple sequence alignment of the predicted amino acid sequence of *S. schenckii *SsSOD and SOD homologues from other fungi. In the alignment, black shading with white letters indicates 100% identity, gray shading with white letters indicates 75-99% identity, gray shading with black letters indicates 50-74% identity. Click here for file

Additional file 2 **Supplementary tables**. Supplemental Table S1 compares SsSOD to other SOD homologues, Supplemental Table S2 compares SsNramp to other Nramp homologues, Supplemental Table S3 compares SsSit to other fungal siderophore transporter homologues and Supplemental Table S4 compares SsGAPDH to other fungal GAPDH homologues. The percent identity of the SsSOD, SsNramp, SsSit and SSGAPDH to other fungal homologues was calculated using iProClass database and the BLAST algorithm. Supplemental Table S5 contains the calculated and expected molecular weights of the proteins identified by co-immunoprecipitation. Click here for file

Additional file 3 **Protein multiple sequence alignment of SsNramp to other fungal Nramp homologues**. Multiple sequence alignment of the predicted amino acid sequence of *S. schenckii *SsNramp and Nramp homologues from various fungi and mouse. In the alignment, black shading with white letters indicates 100% identity, gray shading with white letters indicates 75-99% identity, gray shading with black letters indicates 50-74% identity. The invariant residues are shaded in blue in the consensus line. Bold lines above sequences identify predicted transmembrane helices. Click here for file

Additional file 4 **Protein multiple sequence alignment of SsSit to other fungal Sit homologues**. Multiple sequence alignment of the predicted amino acid sequence of *S. schenckii *SsSit and Sit homologues from various fungi. In the alignment, black shading with white letters indicates 100% identity, gray shading with white letters indicates 75-99% identity, gray shading with black letters indicates 50-74% identity. Bold lines above sequences identify 11 of the possible 13 predicted transmembrane helices. These 11 TM helices were consistently identified by multiple prediction servers. The gray bold lines above sequences identify the two additional TM helices identified by TMHMM. Red boxes highlight motifs that characterize the MFS. Click here for file

Additional file 5 **Protein multiple sequence alignment of SsGAPDH to other fungal GAPDH homologues**. Multiple sequence alignment of the predicted amino acid sequence of *S. schenckii *SsGAPDH and GAPDH homologues from various fungi. In the alignment, black shading with white letters indicates 100% identity, gray shading with white letters indicates 75-99% identity, gray shading with black letters indicates 50-74% identity. Click here for file
