# Correction to: Interaction of the heterotrimeric G protein alpha subunit SSG-1 of *Sporothrix schenckii* with proteins related to stress response and fungal pathogenicity using a yeast two-hybrid assay

**DOI:** 10.1186/s12866-019-1656-7

**Published:** 2019-11-26

**Authors:** Lizaida Pérez-Sánchez, Elizabeth González, Emilee E. Colón-Lorenzo, Waleska González-Velázquez, Ricardo González-Méndez, Nuri Rodríguez-del Valle

**Affiliations:** 1 Department of Microbiology and Medical Zoology, Medical Sciences Campus, University of Puerto Rico, PO Box 365067, San Juan, PR 00936-5067 USA; 2 Department of Radiological Sciences, Medical Sciences Campus, University of Puerto Rico, PO Box 365067, San Juan, PR 00936-5067 USA

**Correction to: BMC Microbiol (2010) 10:317**

**https://doi.org/10.1186/1471-2180-10-317**

Following the publication of this article [1], it was brought to our attention that Fig. 7A lane 2 is identical to Fig. 7B lane 2 and Fig. 7B lane 4 is identical to Fig. 7C lane 4. The corrected Fig. 7 with accompanying figure legend can be seen below. This correction does not change the conclusions drawn from the data.

**Fig. 7:**
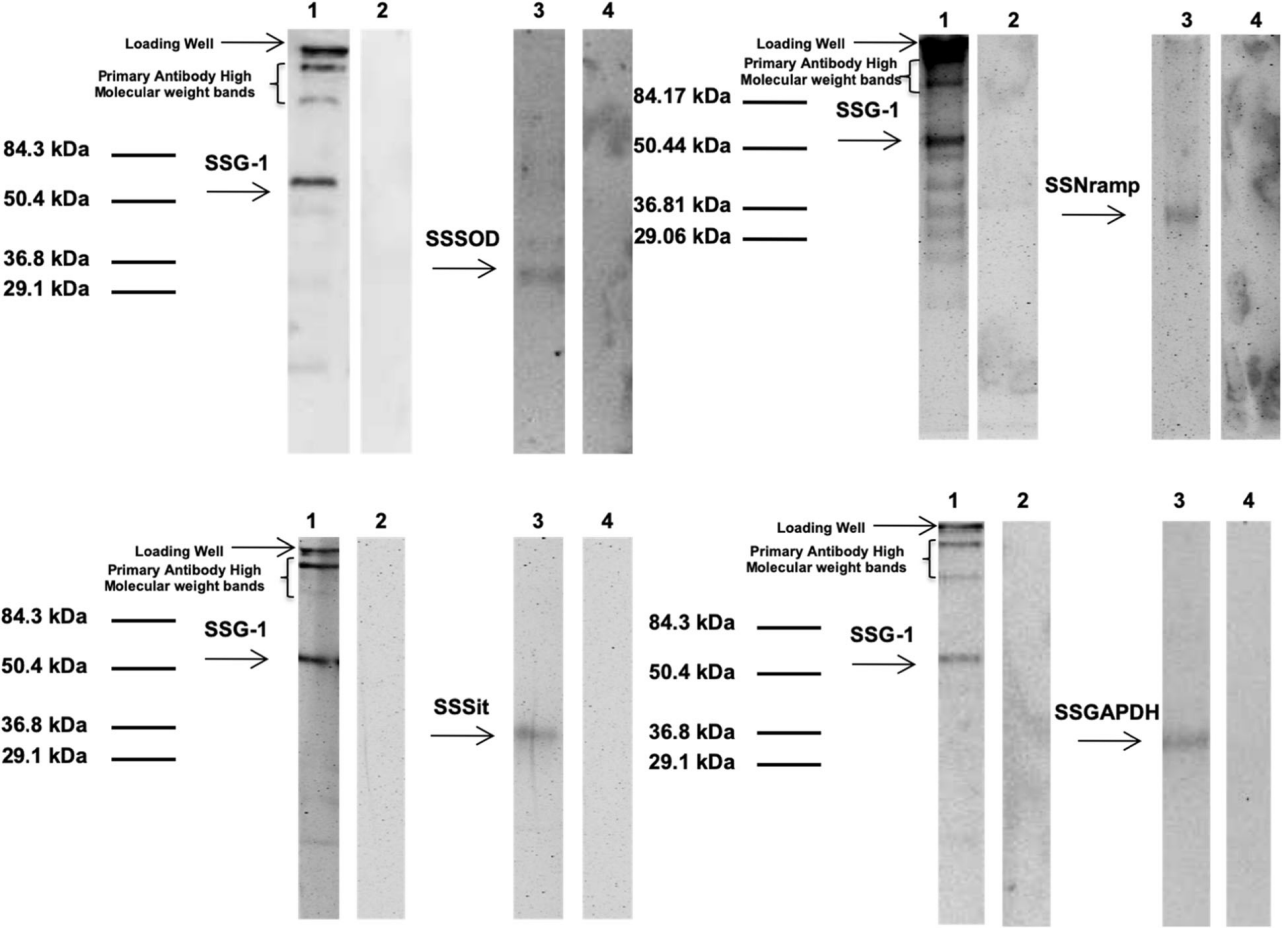
Co-immunoprecipitation and Western Blot analyses of SSG-1 interacting proteins. **a** corresponds to the results of the Co-IP of SSG-1 and SsSOD, **b** corresponds to the results of the Co-IP of SSG-1 and SsNramp, **c** corresponds to the results of the Co-IP of SSG-1 and SsSit and **d** corresponds to the results of the Co-IP of SSG-1 and SsGAPDH. Whole cell free extracts of *S. cerevisiae* cells expressing the complete c-myc tagged SSG-1 coding sequence fused to the GAL4 activation domain (prey protein) and the HA tagged protein fragment fused to the GAL4 DNA binding domain (bait protein) were co-immunoprecipitated as described in Methods. The coimmunoprecipitated proteins were separated using 10% SDS polyacrylamide electrophoresis under non-reducing conditions and transferred to nitrocellulose. Lane 1: Nitrocellulose strips were probed with anti-cMyc antibody as the primary antibody and anti-mouse IgG as the secondary antibody that recognizes both the heavy and light chain of the anti-cMyc (The two high molecular weight bands present belong to the anti-cMyc antibody used for precipitation). Lane 2: Negative controls where no primary antibody was added and probed with the secondary antibody anti-mouse IgG that recognizes both the heavy and light chain of the anti-cMyc antibody. Lane 3: Nitrocellulose strips were probed with anti-HA antibody as the primary antibody and anti-rabbit IgG as the secondary antibody that recognizes both the heavy and light chain of the anti-HA. Lane 4: Negative controls where no primary antibody was added and probed with the secondary antibody anti-rabbit IgG that recognizes both the heavy and light chain of the anti-HA antibody. Pre-stained molecular weight markers were included in outside lanes of the gel. Exposure times to detect the interacting proteins SSSOD using anti-cMyc antibodies and antiHA antibodies were 3.1 s and 9.1 s, respectively. To detect the other binding partners SSNRAMP, SSSIT, and SSGAPDH using anti-cMyc antibody and anti-HA antibody were 1 s for each one
