# Supplementary material for: Interaction of the heterotrimeric G protein alpha subunit SSG-1 of Sporothrix schenckii with proteins related to stress response and fungal pathogenicity using a yeast two-hybrid assay
Source: BMC Microbiol. 2010 Dec 9;10:317. doi: 10.1186/1471-2180-10-317 (PMC3018405; doi:10.1186/1471-2180-10-317)
Supplement: Additional file 1 — Protein multiple sequence alignment of SsSOD to other fungal SOD homologues. Multiple sequence alignment of the predicted amino acid sequence of S. schenckii SsSOD and SOD homologues from other fungi. In the alignment, black shading with white letters indicates 100% identity, gray shading with white letters indicates 75-99% identity, gray shading with black letters indicates 50-74% identity. [file 1471-2180-10-317-S1.PDF]

```
S.sche   1 : MFR--PRLR-----------------------------------------------------------A---PAALGGNITISSSSGASMLARRPAQSALAPIAQ
A.caps   1 : MIP----------------------------------------------------------------QRSLRLR-PGL-------------RAVSSSLSFLRTKP
S.scle   1 : MFR--SRF----------------------------------------------------------------PR-IG----------------LSVQRNF-----
B.fuck   1 : MFR--SRF----------------------------------------------------------------PR-IG----------------RSVQKNF-----
M.gris   1 : MLR--PALR-----------------------------------------------------------I-------P----------------LRRQIQPLRPLV
C.glob   1 : MLR--PGLR-----------------------------------------------------------IAMRPR-VGA----------------PARSTLLPLAC
A.derm   1 : MSGVKPTMQPWLALVGCGWMWLDIGSRKPNRAILAPCQIVGGSGHSWAQTLFPRQWFESVHFPFMILQRSLRPQ-SGL-------------RAVSSSLSFLRTTP
G.zeae   1 : MLR--PRLR-----------------------------------------------------------V---PR-L------------------AFGLGMRPMVN
A.fumi   1 : MSQ--Q---------------------------------------------------------------------------------------------------
H.sapi   1 : MLS--RAVC-------------------------------------------------------------------G----------------TSRQLAPALGYL
             M

S.sche  42 : ARRSIHHLPVSRHDAR-----VGV----PNLLSAEGFDLAWTQHMTLMLNRLNQLAAGTTY-EDR----ELKSIIIRTAGNQSSAAIFNYASMAHNTDFFFKHII
A.caps  28 : QNRSTHYVPQLGHDAA--FKKDGV----PDLLSPQGFDLAWVQYQGFLINKLNLLTAGTPD-ESA----TPGTLLVRYARDANMASVFNYASMAHNNHFFFNCLS
S.scle  18 : IRSFSQKVPPLKHDFT-----KGI----PEFIGPQAFDLSWTAYQKLMVDKLTDATAGIPHLEGR----QPKEIAIMTARDPDQAATFNYASMAFNNNFFFDCIQ
B.fuck  18 : IRSISQHIPPLKHDFS-----NGI----PEFIGPQAFELAWTSYQKLMVDKLSDAIAGVPHLDGK----QPKDIALLCARDPDQAATFNYASMAFNNNFFFNCLQ
M.gris  22 : QRRFEHLMPVLQKDKQQ-FLEHGIGPEDDQFLSARAVDLAWTQYMSLALKKLNRLTNGKEN-DVSTGRDSLRDLAIRTARDPLNAPTFNYASMAHNNHFFFDGLS
C.glob  28 : QARSMHALAPLSYDLKGEATQNGI----SDFLSPAAFNISWTQYQTYLLQKLNILTAETDY-ESH----QIKDILLGTARDPGSAPIFNHASMAHNNHFFFKHIS
A.derm  92 : QTRSTHYVPQLGHDAS--FKQHGV----PDLLSPQGFDLSWVQYQSFLINKLNLLTAGTPD-ESA----TPGTLLVRYARDASMASVFNYASMAHNNHFFFNCLS
G.zeae  23 : VRRSIHSVPELPHDYS-----QGV----PNLMSSGGFSLAWTQYMTLMVEKLNALTVGTEL-EDK----DTKTIALMTAREPNQAPIFNYASMAHNNHFFFQGIS
A.fumi   5 : -----YTLPPLPYPYD------AL----QPYISQQIMELHHKKHHQTYVNGLNAALE-AQK-KAAE----ANDVPKLVSV--QQAIKFNGGGH-INHSLFWKNLA
H.sapi  21 : GSRQKHSLPDLPYDYG------AL----EPHINAQIMQLHHSKHHAAYVNNLNVTEE-KYQ-EALA----KGDVTAQIAL--QPALKFNGGGH-INHSIFWTNLS
                     p l  d        g               l w          Ln                         a     A  FN asma N  fFf

S.sche 133 : PASASPAEAAAAREIP-ATLRLAIEDNFGSVETLRREFLAIAQGMFGPGFIWLVKANGLNQMGRGGDSLRLLNTYHAGSPYPGAHYRRQMTDMNTVGAEVAEN--
A.caps 122 : PQHV---------PIP-EHLSKSIEDSCSSVESLKAEFLATANAMFGPGFVWLVKSKDTG-------QLKILTTYIAGSPYPAAHFRRQPVDMATQTTGITGG--
S.scle 110 : PNPASEP------EMS-ERLRVAIEASFSSVDSLKKEFVITASKMFGPGFVWLMKDRF--------DSLSLLTTYLAGSPFPGAHYRKQTKDMNTESENLTDY--
B.fuck 110 : PNPASEP------VMS-EKLRAAIESSFSSVDGLKKEFVITASKMFGPGFVWLMKDRH--------DRLSLMTTYLAGSPFPGAHHRRQPKDMNTESESVTDY--
M.gris 125 : SEPV---------EMP-KLLREALEQDFGSIETLQREFLVTALAMFGPGFVWLVKTKTTA----GRDVFRLLPTYLAGTPYPKAHYRRQPVDMNTADSQGPEANS
C.glob 124 : PKPV---------EVP-EPLRSRLEQSFGSMDTLRQEMVYTAAGMFGPGFVWLVKTSQPG----LPVSFKVLTTYAAGSPYPAAHWRRQEHDMNTAAGSGTEAGI
A.derm 186 : PEQV---------PIP-EHLSKSIEDSCSSVESLKAEFLATANAMFGPGFVWLVKSKDTG-------QLKILSTYIAGSPYPAAHFRRQPVDMATQTTGITGG--
G.zeae 114 : PTGT---------PMP-DALRSELEASFSSIETLRREFVITASAMFGPGFLWLVKAGP--------GDYRLLPTYLAGSPYPGAHWRAQSTDMNTLGKDGSA---
A.fumi  86 : PEKSGGG----KIDQA-PVLKAAIEQRWGSFDKFKDAFNTTLLGIQGSGWGWLVTDGPKG-------KLDITTTH-DQDPV------------------------
H.sapi 107 : PNGGG---------EPKGELLEAIKRDFGSFDKFKEKLTAASVGVQGSGWGWLGFNKERG-------HLQIAACP-NQDPL------------------------
             p                  L    e    S   l  ef  ta  mfGpGf WL k                  ty ag P p ah r q  dm t

S.sche 235 : ------EDDPAENWL-KRQAVAAD--PSLWKQPDRRPPGGV---EAIPLLCVSTWEHVWLRDYGLGAD--GYGGKAAFVEAWWNAIDWEAVASLANLNRQ-----
A.caps 208 : ----ENLEQIRKL-T-PTNRVGSM--GA-YSSQKFMAPGAI---DIHPILCVNTWEHVWLPDWGV-------GGKAQFLESWWNRINWDEVAHNSSQAGPI----
S.scle 198 : ------HRQRLAG-P-PVNTVGAH--G-ALSK-ERKPPGGI---EQTPILCVNTWEHVWIPDYGMGVD--NVGGKKVYVENWWNVIDWSVVADNAALSGNSI---
B.fuck 198 : ------YRQKLAA-P-PVNTVGAH--G-HLSK-ERKPPGGI---EATPILCVNTWEHVWIADYGMGTD--DVGGKKVYVENWWNVIDWNVVADLAALSGNSA---
M.gris 216 : VDGWMNKQQAA---------VSSD--GS-LEKT-EKANGGISETSLTPVLCLNTWEHVWTTDYGIVYGEKERESKRLYVSRWWKHIDWDVVSSLANIR-------
C.glob 215 : ATGRAYLDQTAYG-TGSSSRWGSGGPGSATARRVAHAPGGT---DLVPVLCINTWEHVWLWDYGFGVGAP-DRGKLGYAEKWWKHVDWELVQKEADLQRLEMSAN
A.derm 272 : ----ENLEQIRKL-T-PTNRVGSM--GA-YSSQKFMAPGAI---DIHPILCVNTWEHVWLPDWGV-------GGKPQFLEAWWNRINWDEVAHNASQAGPI----
G.zeae 198 : -----------RNFI-HNQAYG------ASKRSDNLPPGGI---ELEPLLCLNTWEHAWLLDWGVGAG--GQGGKVAFAESWWELIDWEKVAQKSGVLRPDFKSA
A.fumi 154 : ----------------------------------------T---GAAPVFGVDMWEHAYYLQYL--------NDKASYAKGIWNVINWAEAENRYI-AGD-----
H.sapi 171 : -------------------------------------QGTT---GLIPLLGIDVWEHAYYLQYK--------NVRPDYLKAIWNVINWENVTERYM---------
                                                   g        P lc  tWEH w  d g          k      wW  i W  v
```

```
S.sche 321 : --------------------------------------------------------------KLKT
A.caps 290 : -------------------------------------------STRAG-----TAR-SSYRRGARF
S.scle 283 : ------------------------------------------------------------VRFERE
B.fuck 283 : ------------------------------------------------------------SKLERN
M.gris 301 : -----------------------------------------------------------PRPEPKL
C.glob 315 : AGAP--------------------------------------APSLSS-----PSL-SSPAPVPRA
A.derm 354 : -------------------------------------------STRPG-----TAR-SSYRRGARF
G.zeae 280 : IETKRRRASEFYQPSAQSKKVKHQKEKTYNTRYSLVVTDPTTNPALRGLTMGERTGPRILHELWSD
A.fumi 202 : ---------------------------------------------------------KGGHPFMKL
H.sapi 219 : --------------------------------------------------------------ACKK
```
