# Supplementary material for: Interaction of the heterotrimeric G protein alpha subunit SSG-1 of Sporothrix schenckii with proteins related to stress response and fungal pathogenicity using a yeast two-hybrid assay
Source: BMC Microbiol. 2010 Dec 9;10:317. doi: 10.1186/1471-2180-10-317 (PMC3018405; doi:10.1186/1471-2180-10-317)
Supplement: Additional file 2 — Supplementary tables. Supplemental Table S1 compares SsSOD to other SOD homologues, Supplemental Table S2 compares SsNramp to other Nramp homologues, Supplemental Table S3 compares SsSit to other fungal siderophore transporter homologues and Supplemental Table S4 compares SsGAPDH to other fungal GAPDH homologues. The percent identity of the SsSOD, SsNramp, SsSit and SSGAPDH to other fungal homologues was calculated using iProClass database and the BLAST algorithm. Supplemental Table S5 contains the calculated and expected molecular weights of the proteins identified by co-immunoprecipitation. [file 1471-2180-10-317-S2.DOC]

| **Supplemental Table S1: Comparison of *S. schenckii* SsSOD to SOD homologues** | | |
| --- | --- | --- |
| Database_Accession number | Organism | Percent Identity |
| GenBank_ABF46644.3 | *Sporothrix schenckii* | 100 |
| UniRef100_C0NXL0 | *Ajellomyces capsulata* | 43 |
| UniRef100_D1Z7E6 | *Sordaria macrospora* | 39 |
| UniRef100_Q2HFL4 | *Chaetomium globosum* | 42 |
| UniRef100_C7YQ45 | *Nectria haematococca* | 47 |
| UniRef100_B2AYC2 | *Podospora anserina* | 35 |
| UniRef100_UPI000023DF06 | *Gibberella zeae* | 48 |
| UniRef100_A4RF21 | *Magnaporthe grisea* | 44 |
| UniRef100_Q92450 | *Aspergillus fumigatus* | 23 |
| UniRef100_P04179 | *Homo sapiens* | 32 |

Analysis was carried out using the iProClass database, the BLAST algorithm and the PIR pairwise alignment tool. All searches in the PIR database were done with the submitted sequence at GenBank.

| **Supplemental Table S2: Comparison of *S. schenckii* SsNramp to Nramp homologues** | | |
| --- | --- | --- |
| Database_Accession number | Organism | Percent Identity |
| GenBank_ACV31218.1 | *Sporothrix schenckii* | 100 |
| UniRef100_A7E580 | *Sclerotinia sclerotiorum* | 54 |
| UniRef100_Q7S317 | *Neurospora crassa* | 56 |
| UniRef100_P38925 | *Saccharomyces cerevisiae* | 47 |
| UniRef100_C5FMY4 | *Nannizzia otae* | 52 |
| UniRef100_A6RJN0 | *Botryotinia fuckeliana* | 59 |
| UniRef100_C5PGA8 | *Coccidioides posadasii* | 51 |
| UniRef100_B6HQ64 | *Penicillium chrysogenum* | 49 |
| UniRef100_B0Y6T3 | *Aspergillus fumigatus* | 48 |
| UniRef100_P49282 | *Mus musculus* | 29 |

Analysis was carried out using the iProClass database, the BLAST algorithm and the PIR pairwise alignment tool. All searches in the PIR database were done with the submitted sequence at GenBank.

| **Supplemental Table S3: Comparison of *S. schenckii* SsSIT to other fungal siderophore-iron transporter homologues** | | |
| --- | --- | --- |
| Database_Accession number | Organism | Percent Identity |
| GenBank_ACV31217.1 | *Sporothrix schenckii* | 100 |
| UniRef100_UPI000023F6D4 | *Gibberella zeae* | 74 |
| UniRef100_Q2HC00 | *Chaetomium globosum* | 67 |
| UniRef100_Q0V5Z9 | *Phaeosphaeria nodorum* | 54 |
| UniRef100_Q2UFX6 | *Aspergillus oryzae* | 48 |
| UniRef100_B8NHW7 | *Aspergillus flavus* | 46 |
| UniRef100_A2R1X7 | *Gibberella moniliformis* | 25 |
| UniRef100_Q92341 | *Schizosaccharomyces pombe* | 36 |
| UniRef100_Q5KMV2 | *Cryptococcus neoformans* | 33 |
| UniRef100_C1GDN7 | *Paracoccidioides brasiliensis* | 29 |

Analysis was carried out using the iProClass database, the BLAST algorithm and the PIR pairwise alignment tool. All searches in the PIR database were done with the submitted sequence at GenBank.

| **Supplemental Table S4: Comparison of *S. schenckii* SsGAPDH to GAPDH fungal homologues** | | |
| --- | --- | --- |
| Database_Accession number | Organism | Percent Identity |
| GenBank_ACY38586.1 | *Sporothrix schenckii* | 100 |
| UniRef100_P32637 | *Podospora anserina* | 85 |
| UniRef100_Q6PN65 | *Chaetomium globosum* | 86 |
| UniRef100_Q6B521 | *Beauveria bassiana* | 87 |
| UniRef100_UPI000023F2B8 | *Gibberella zeae* | 84 |
| UniRef100_Q5EMS5 | *Magnaporthe grisea* | 84 |
| UniRef100_C1G5F6 | *Paracoccidioides brasiliensis* | 78 |
| UniRef100_P54118 | *Neurospora crassa* | 82 |
| UniRef100_Q8WZN0 | *Sordaria macrospora* | 82 |
| UniRef100_P04406 | *Homo sapiens* | 71 |

Analysis was carried out using the iProClass database, the BLAST algorithm and the PIR pairwise alignment tool. All searches in the PIR database were done with the submitted sequence at GenBank.

**Supplemental Table S5: Calculated and expected molecular weights for the proteins expressed in the yeast two-hybrid experiment.**

| Protein | MWt of prey protein fragment (kDa) | MW of GAL-4 domain* (kDa) | MW of prey protein fragment+Gal-4 domain (kDa) |
| --- | --- | --- | --- |
| SsSOD | 7.069 | 18-24 | 33.5 (expected 25.01-31.01) |
| SsNramp | 17.635 | 18-24 | 35.5 (expected 35.6-41.6) |
| SsSIT | 11.04 | 18-24 | 33.2 (expected 29.04-35.04) |
| SsGAPDH | 15.3 | 18-24 | 35.5 (expected 33.3-39.3) |

* This is the molecular weight range that the manufacturer suggests for the GAL-4 domain
