# Supplementary material for: Interaction of the heterotrimeric G protein alpha subunit SSG-1 of Sporothrix schenckii with proteins related to stress response and fungal pathogenicity using a yeast two-hybrid assay
Source: BMC Microbiol. 2010 Dec 9;10:317. doi: 10.1186/1471-2180-10-317 (PMC3018405; doi:10.1186/1471-2180-10-317)
Supplement: Additional file 3 — Protein multiple sequence alignment of SsNramp to other fungal Nramp homologues. Multiple sequence alignment of the predicted amino acid sequence of S. schenckii SsNramp and Nramp homologues from various fungi and mouse. In the alignment, black shading with white letters indicates 100% identity, gray shading with white letters indicates 75-99% identity, gray shading with black letters indicates 50-74% identity. The invariant residues are shaded in blue in the consensus line. Bold lines above sequences identify predicted transmembrane helices. [file 1471-2180-10-317-S3.PDF]

```
S.sche    1 : MN-RPSRTDEPPRSDGLNQSPNELSNDLTTNEDLNGTANSRRFRRSVSPHDLQAGLPGETNRFGI------AVDNPDDPGY--Q----KTAAAGPITTRV
S.scle    1 : MN-CPSRTDEPLEGDGYNQNPNALANDLTTREDLNGRANGRALRRSLED-EGVESINDLVDDTGDGKVTGGENGAGGGAGGERG----GGGGGRGGDGKS
N.cras    1 : MCASASRTDEPRKSQGFNQSPNPLSADLTTNQDLDGIVNQRANRTGS----GGTGISAQLRQDGV------GDDGGGLPRPERD----RPPGGDMIKTPS
C.posa    1 : MN-CPSRTDNPSDNPGWNQSPPLLTADLTTRKDLNGMANMRVRKGKGT---GPNGTELNPGQDIPS---KSTDEDEPGAHSAGK----DAHASTVGEREV
P.chry    1 : MN-CPSRTDDTLENPSWNQSPPPFSPDITTRNDFNGLANSRVHRRHAN---S-ANAAGDDPLSIDV---RPNSQQDRDPGK-E-----KVPSG-------
A.fumi    1 : MN-CPSRTDDTLKHPGWNQNPPALNADMTTRNDLNGIANSKVHRRHAS---GMGGVSGEGIMETDS---QSHNQDCTESEIVET----KT-LG-------
B.fuck    1 : MI-WWR-ILEMGR--------------------------RRGVRRVLE--GGLGGIGAVGL-----------------------------EGGSGGDGKS
N.otae    1 : MN-KTSRTDEPFEGEGYNQNPNPLSNDLITNEGLNGIVNSRELKREEP--QSLHGITQQADRDKT------SSI---APEMRRGDAFNLLAKRWIGQLSR
S.cere    1 : MVN-VGPSHAAVA----------VDASEARKRNISEEVFEL---------------RDKK------------------------D------S-----TVV
M.musc    1 : MV-LDPKEKMPDD----------------------------------------------------------------------------GASGDHGDSAS
              M

                                                                            TMS1
S.sche   88 : D--S-VASARRPSQSSVPPNSDGGALQAGGHGDG--NR-RQGPFWKVVSSLRTAIRFVGPGFIVSVAY---------------------IDPGNYSTDIA
S.scle   95 : G--H-VVGP-TVEALDIRDQSGTKRGSE--RSCV--NI-SKEQLVKMKQVMVKFAKFVGPGFMVAVAY---------------------IDPGNYATDVA
N.cras   87 : S--T-VSSP-TNPQPSPPPSSNQVEAPS--PGSV--KQ-AKSRLGQIKHALITFGQFVGPGVMISVAY---------------------IDPGNYATDIA
C.posa   90 : GLEEDVDN-RRISSSSWPNFHGYSGGAG--SGNLG-KFGPRELIHRLVKTLRKFGQFIGPGFLIAVAY---------------------IDPGNYATDVA
P.chry   80 : ----EVIA-ADSGGDTPPRSSG-SPRH----PFY--TSL-TSYSLHCARTLAKFSRFIGPGFLIAVAY---------------------IDPGNYATDVA
A.fumi   82 : ----GVIA-AASGYENPQSHRE-SPRRY--TNLT--TII-TSHIIHVRQSLAKFARFVGPGFLIAVAY---------------------IDPGNYATDVA
B.fuck   42 : G--H-VVGP-SVETLDIRDQSRRGRGAECGRSFL--HV-SKEQLVKMKQVMVKFAKFVGPGFMVAVAY---------------------IDPGNYATDVA
N.otae   89 : G--G-DKSP-LSDTATITPGDGSTG--S--CDQG--NG-PQSLIERFKNGLLKFCSFIGPGFMVSVAYSMESFHSQIRYFSELTLSLLSVDPGNYATGIA
S.cere   40 : ------IE-GE--APV-RTFTSSSSNHE--RED-T-YV-SKRQV--MRDIFAKYLKFIGPGLMVSVAY---------------------IDPGNYSTAVD
M.musc   24 : L--G-AINP-AYSNSSLPHSTGDSEEPFTT--YFDEKI-PIPEEEYSCFSFRKLWAFTGPGFLMSIAY---------------------LDPGNIESDLQ
                                                                   k   F GPGf   vAY                     iDPGNy td a

                          TMS2                                                   TMS3                         TMS4
S.sche  161 : AGASYRYKLLFVVLMSNCFAIYLQSMCIKLGTVSGRNLAAACRAFLPRWLNISLYILAEVAIIATDIAEVIGTAIALNLLQP-KIPLVAGCTISIVDVFI
S.scle  165 : AGATYRFKLLFIVLMSNIFAIFLQSLCIKLGTVSGLNLAEACRAFLPKWLNIGLYILAEGAIIATDIAEVIGTAIAINLLIP-QIPLVAGCALSILDVLI
N.cras  157 : AGASYRFKLLFIVLLSNLFAILLQSLAIKLGTVTGLDLSSACRAFLPRWLNYFIYALAEIAIIATDIAEVIGTAIAFNLLSP-KIPLVAGCALSIIDVML
C.posa  165 : AGASAKYSLLFIIFMSNVIAVFLQSLCIKLGSVTGLNLAENCKAHLPKWLNILLYVLSEAAIIATDIAEVVGSAISLNLLF--NIPLVAGCAITLVDVMV
P.chry  146 : AGADFKYALLFIVLLSNLFAILLQSLCIKLGSVTGLNLAENCREHLPRWLVILLYIMAEAAIIATDIAEVVGSAIALNLLL--NIPLVAGCAITLVDVLF
A.fumi  150 : AGAEFRYALLFIVLLSNLFAIFLQSLCIKLGSVTGLNLAENCREHLPKWLVYILYFLSEAAIVATDIAEVVGSAIALNLLL--KIPLVAGCAITLADVLF
B.fuck  114 : AGATYRFKLLFIVLMSNIFAIFLQSLCIKLGTVSGLNLAEACRAFLPKWMNYALYILAEGAIIATDIAEVIGTAIAINLLIP-QIPLVAGCALSILDVLI
N.otae  178 : AGASYRFRLLFVILMANLFAILLQSLAVKLGTVSGLNLAEACRAFLPRWLNILLYVLAEVAIIATDIAEVIGFAIGLNLLIP-KVPLVAGCAISIFDVMI
S.cere  102 : AGASNQFSLLCIILLSNFIAIFLQCLCIKLGSVTGLDLSRACREYLPRWLNWTLYFFAECAVIATDIAEVIGTAIALNILI--KVPLPAGVAITVVDVFL
M.musc   96 : SGAVAGFKLLWVLLLATIVGLLLQRLAARLGVVTGLHLAEVCHRQYPKVPRIILWLMVELAIIGSDMQEVIGSAIAINLLSAGRVPLWGGVLITIADT-F
              aGA     LLf  l sn  ai LQsl ikLG V Gl La  Cr  lP wl   ly   E AiiatDiaEV G AIa NlL     PLvaGca    Dv
```

```
                                  TMS5                                              TMS6
S.sche 260 : VLLFCKPENGTRSGLRAFELIVVPLVLGVVICFCIQLSMIDHTTTSVGEVFRG-YLPSAAVIEQQG---LYQACGILGATVMPHSLYLGSGIVQARLREY
S.scle 264 : LLAFYPG-NGEMKRLRYFEMFVVALVLGVVICFCIQLSLIQ-D-TSVGQVFKG-YLPSQAIVQSKG---LYQACGILGATVMPHSLYLGSGIVQPRLLDY
N.cras 256 : ILVFYNP-NGQMKGLRIFEFFVCILVMGVVACFCIQLSMIS-N-TSVGEVFKG-YLPSSAVIEQQG---LYQACGILGATVMPHSLFLGSGIVQPRLRAY
C.posa 263 : ILIFYKP-HGTMRALRAFEFFVMALVFGVVGCFCVQLSLIK-E-SSVGEIFKG-YLPSEAIVKDNGAVSLYLSCGILGATVMPHSIFLGSGVVQSRLREY
P.chry 244 : ILIFYRP-NGKMWGLRLFEFFVMALVLGVVVCFCIQLSLIK-D-QSVGEVFRG-YLPSSAIVQSEG---LYQSCGILGATVMPHSLFLGSGVVQSRLKEF
A.fumi 248 : LLIFYRP-NGSIWGLRLFEYFVMALVLGVVVCFCIQLSLIR-E-QSVRDVFRG-YLPSSAIVQSKG---LYQSCGILGATVMPHSIFLGSGVVQPRLKEF
B.fuck 213 : LLIFYPG-NGEMKRLRYFEIFVAALVLGVVVCFCIQLSLIK-D-TSVGEVFKG-YLPSKAIVEQKG---LYQACGILGATVMPHSLYLGSGIVQPRLLDY
N.otae 277 : ILFFYRP-DGSMKALRAFEAVIICLVFGVVICFCIQLSLIK-N-ASVGQVFRG-YLPSGELVESQA---IYQACGILGATVMPHSLYLGSGIVQPRLREY
S.cere 200 : IMFTYKPGASSIRFIRIFECFVAVLVVGVCICFAIELAYIPKS-TSVKQVFRG-FVPSAQMFDHNG---IYTAISILGATVMPHSLFLGSALVQPRLLDY
M.musc 195 : VFLFLDKYG--LRKLEAFFGFLIT-IMALT--FGYEYITV-K-PSQ-SQVLRGMFVPSCPGCRTPQ---VEQAVGIVGAVIMPHNMYLHSALVKSRQVNR
              l fy    g    lr Fe fv  lv gvv cFciqls i     sv  vf G ylPS       g    yq cgIlGAtvMPHs  LgSg Vq Rl

                                                                                                 TMS7
S.sche 356 : DEKYGLLPPEEDQQPQMVEREADDEASDNDNGRHGNRTLSSLRQQFYLAKKHTEDRSQTHTYIPSLRAIRHCYKYSVVEVAVSLFTYALF--VNSAIL-I
S.scle 357 : DKKNNFVKKDFDL--------------G-----------------------------TKEPYYPSLPAIKFCLKYSYAELAICLFTFALF--VNSAIL-I
N.cras 349 : DEQRGLLPAEPVSA------N-----SSDTN------S----------------DYADKVHYVPSQSAIKHSLKYSIAELALSLFTFALF--VNSAIL-I
C.posa 359 : DINRGYIDPSVQY--------------G--------------------------NTY-EDKYRPTVHAIRGCLKYSVLELVLSLFTFALF--VNSSIL-I
P.chry 337 : DVNCGYVETSVPL--------------G--------------------------NAEGEVKYRPSIYAIRGCMKYSIIELSLSLFTFALF--VNSAIL-I
A.fumi 341 : DVTKGYVDPAVCL--------------G--------------------------STDGKVEYRPSIHAIRGCMKYSIIELSLSLFTFALF--VNSSIL-I
B.fuck 306 : DKQNNIVKKDFDL--------------G-----------------------------TKETYYPSLPAIKFCLKYSYAELAICLFTFALF--VNSAIL-I
N.otae 370 : DAKSKLLPRELMSA------S-----SSITE------G----------------VDKDKLHYIPSIRAIRHSLKVSITELTVSLFSFALF--VNSAIL-I
S.cere 295 : DVKHGNYTVSEEQD--KVKKS-----KS------TE-E---------------IMEEKYFNYRPTNAAIKYCMKYSMVELSITLFTLALF--VNCAIL-V
M.musc 284 : ANKQEV--------------R-----EA-------N-K------------Y---------FFIESCIAL-F-VSFIINVFVVSVFAEAFFEKTNKQVVEV
             d                                                               y ps  Ai    kys  el   lFt AlF  vNs il i

                                     TMS8                                              TMS9
S.sche 453 : VAG-AALYQNT-----IAMGADIFAVHELLSNTLSKAAGFVFALALLLSGLSAGVVCTVAGQMVCEGALQWTIAPWLRRLLTRSISILPSIVIAGAVGRD
S.scle 411 : VAG-AALYNNP-----AAPDADLFGIYDLLVSSISKGAGTIFALALLLSGMSAGIVCTIAGQMVSEGALNWTIRPWLRRLLTRSLSITPSIIIAGAVGRE
N.cras 413 : VAG-ASLYQNP-----TALDADIFAIHSLLSSSISPAAGTIFALALLLSGVSAGIVCTIAGQMVSEGALRWKMRPWLRRLITRSISITPSILIAAVVGKD
C.posa 415 : VAG-ASLSGSE-----ASD-ADLFGIHRLLSETISPAAGTVFAIALLLSGISAGIVCTIAGQMVSEGMLNWSVAPWLRRFITRSISIIPSVIIAAAVGKE
P.chry 394 : VAG-AALYDVP-----GGGKADLFGIHSLLSKSIAPAAGLVFALALLLSGISAGIVCTIAGQMVSEGMLNWSIRPWLRRLITRSISIIPSIIIAAAVGKE
A.fumi 398 : VAG-ASLYGSS-----GADNADLWGIHDLLSSSIAPVAGLIFALALLLSGISAGIVCTMAGQMVSEGMLKWTIRPWLRRLITRSISIIPSIIIAAAVGKD
B.fuck 360 : VAG-SALYNNP-----AAPEADLFGIYDLLVSSISKGAGTIFALALLLSGTSAGIVCTIAGQMVSEGALNWTIRPWLRRLVTRSLSITPSIIIAGAVGRE
N.otae 434 : IAG-ASLYQNK-----DALEADIFGIHALLSKSISPAVGTIFALALLLSGISAGVVCTIAGQMVSEGALNWKIRPWLRRLVTRSISIIPSIIIAGAVGRQ
S.cere 363 : VAG-STLYNSP-----EADGADLFTIHELLSRNLAPAAGTIFMLALLLSGQSAGVVCTMSGQIVSEGHINWKLQPWQRRLATRCISIIPCLVISICIGRE
M.musc 334 : CKNNSSPHADLFPSDNSTLAVDIYKGGVVLGCYFGPAALYIWAVGILAACQSSTMTGTYSGQFVMEGFLNLKWSRFARVILTRSIAIIPTLLVAVFQDVE
             vag    ly          aD  f  i   lL        ag  falalLlsG Sag  vcT aGQmVsEG l  w    pwlRrl TRsisI Ps   ia  vg
```

```
                          TMS10
S.sche 547 : GLDAALNASQVVLSIVLPFVTAPLLWFTSFDKYMTVQPGA-A-RFALRVRYRKTASSTSD-IG-NVNQNQGTPDGEEGDPRGNEAPAKMANSWPTA--I-
S.scle 505 : GLSAALNASQVALSIVLPFVTAPLIYFTCRNKFMTVRCQNR-NSFAEDDENTRGNVDGPMEIE-SVYEN---DGTGMSGETGE--GTKMRNHWITA--V-
N.cras 507 : GLNAALQGSQVALSVVLPFVTAPLIWFTSRDRYMMVMPGG-A-RYQVEEEEAVGDKRRLFSAK-RGWLG---RKMGNSNNGGEEGGVKMANSWLTT--I-
C.posa 508 : GLNAALTASQVVLSAMLPFVSAPLIYFTSRSRYMTVE------A----G---LM-------------------TGNSNPEGQEVEAVSMKNNWLVT--I-
P.chry 488 : GIDRTLTASQVALSVILPFVSAPLIWFTCLNRYMTVR------T----E---EA-------------------SGQEG--EVHVVTVPMRNNLLTS--V-
A.fumi 492 : GLDKTLTASQVVLSVILPFVTAPLIYFTCRNRYMTVP------A----D---RV-------------------GGEEA--DNLSEGVKMRNNFLVS--A-
B.fuck 454 : GLSAALNASQVALSIVLPFVTAPLIYFTCRNKYMTVRCQGRPNAFGNDDENARGNGDATMESE-SVYEH---EGAGLGVDMGE--GTKMRNHWITA--A-
N.otae 528 : GLNATLNASQVVLSVVLPFVAAPLIYFTCLSKYMTVQ------P----G----------------------------------------------A--I-
S.cere 457 : ALSKALNASQVVLSIVLPFLVAPLIFFTCKKSIMKTEITVD--HTEE--DS-HNHQNNNDRSAGSVIEQDGS-SGMEIENGKDVKIVYMANNWIIT--V-
M.musc 434 : HLTGMNDFLNVLQSLQLPFALIPILTFTSLRPVMSEFSN--------------GI-------------------GWRIAGGILVLIVCSINMYFVVVYVQ
             gl   l asqV lS  LPFv aPli FT     M v                                                    m n

                       TMS11
S.sche 640 : -LG-LAIWIFITVLNIANLVLL-----------GKG-------------------------------N
S.scle 595 : -VA-AVIWTIMAAMNVANLVLL-----------GKGEV------------------------------
N.cras 598 : -VA-GLVWGLMAVMNVANLVLL-----------GKGN------------------------------S
C.posa 573 : -LG-VLIWIVITVLNIALLVLV-----------GMGK------------------------------A
P.chry 551 : -VA-VLVWIIIVVMNVALLVLV-----------GMGKA-----------------------------S
A.fumi 555 : -LA-LLIWLVIAVMNVALLVLV-----------GLGKA------------------------------
B.fuck 545 : -VA-GVIWTIMAAMNVANLVLL-----------GKGDG-----------------------------V
N.otae 569 : -IA-VLVWLFITVMNIANLVLL-----------GQGA-------------------------------
S.cere 548 : -IA-IIVWLFLSLLNVYAIVQL-----------GMSHG---DI------------------------S
M.musc 501 : ELGHVALYVVAAVVSVAYLTFVFYLGWQCLIALGLSFLDCGRSYRLGLTAQPELYLLNTVDADSVVSR
                    w      n a lvl            G g
```
