# Supplementary material for: Interaction of the heterotrimeric G protein alpha subunit SSG-1 of Sporothrix schenckii with proteins related to stress response and fungal pathogenicity using a yeast two-hybrid assay
Source: BMC Microbiol. 2010 Dec 9;10:317. doi: 10.1186/1471-2180-10-317 (PMC3018405; doi:10.1186/1471-2180-10-317)
Supplement: Additional file 4 — Protein multiple sequence alignment of SsSit to other fungal Sit homologues. Multiple sequence alignment of the predicted amino acid sequence of S. schenckii SsSit and Sit homologues from various fungi. In the alignment, black shading with white letters indicates 100% identity, gray shading with white letters indicates 75-99% identity, gray shading with black letters indicates 50-74% identity. Bold lines above sequences identify 11 of the possible 13 predicted transmembrane helices. These 11 TM helices were consistently identified by multiple prediction servers. The gray bold lines above sequences identify the two additional TM helices identified by TMHMM. Red boxes highlight motifs that characterize the MFS. [file 1471-2180-10-317-S4.PDF]

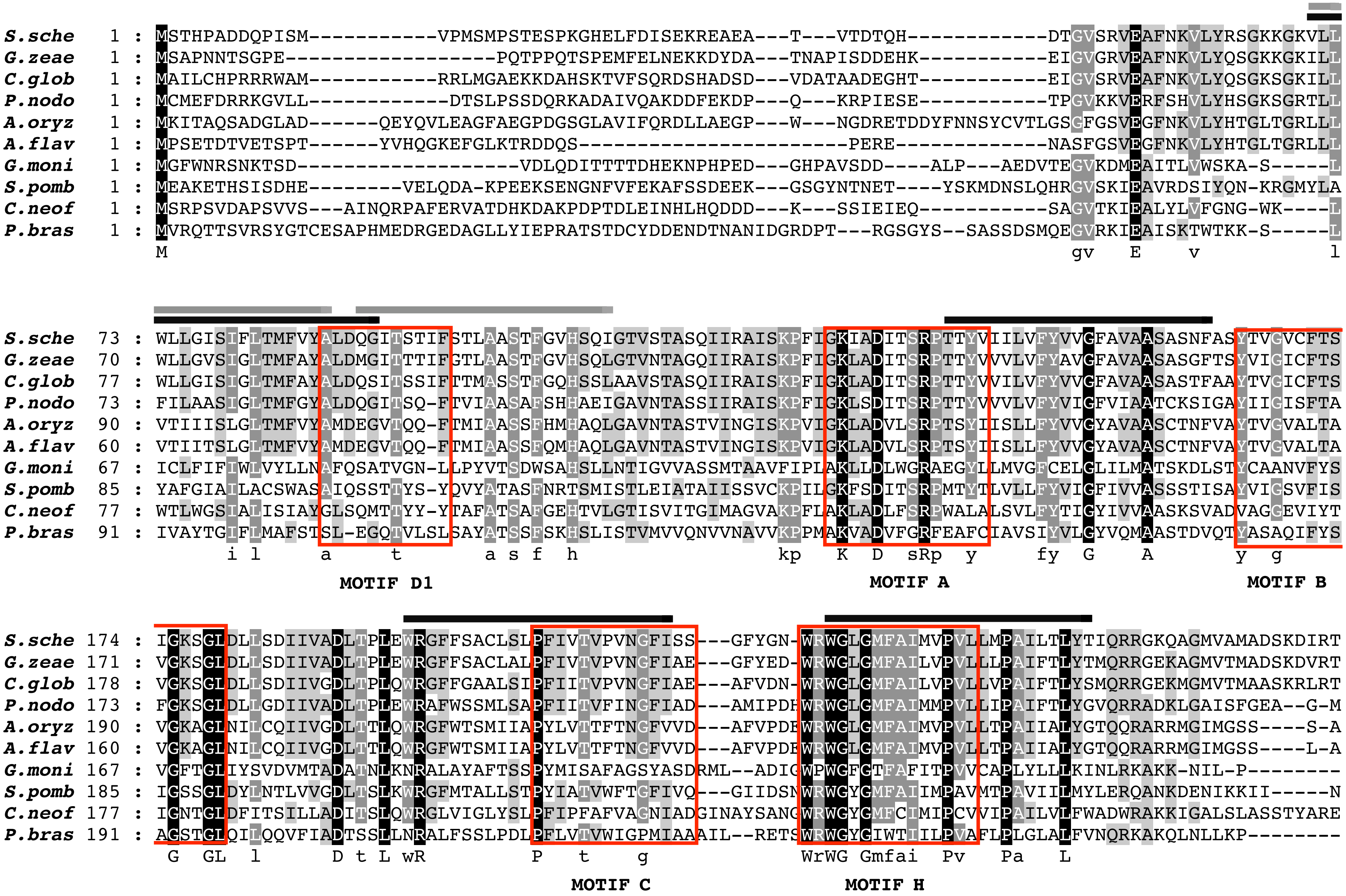

S.sche 1 : MSTHPADDQPISM-----------VPMSMPSTESPKGHELFDISEKREAEA---T---VTDTQH------------DTGVSRVEAFNKVLYRSGKKGKVLL
G.zeae 1 : MSAPNNTSGPE-------------------PQTPPQTSPEMFELNEKKDYDA---TNAPISDDEHK-----------EIGVGRVEAFNKVLYQSGKKGKILL
C.glob 1 : MAILCHPRRRWAM-----------RRLMGAEKKDAHSKTVFSQRDSHADSD---VDATAADEGHT-----------EIGVSRVEAFNKVLYQSGKSGKILL
P.nodo 1 : MCMEFDRRKGVLL------------DTSLPSSDQRKADAIVQAKDDFEKDP---Q---KRPIESE-----------TPGVKKVERFSHVLYHSGKSGRTLL
A.oryz 1 : MKITAQSADGLAD------QEYQVLEAGFAEGPDGSGLAVIFQRDLLAEGP---W---NGDRETDDYFNNSYCVTLGSGFGSVEGFNKVLYHTGLTGRLLL
A.flav 1 : MPSETDTVETSPT------YVHQGKEFGLKTRDDQS-----------------------PERE-------------NASFGSVEGFNKVLYHTGLTGRLLL
G.moni 1 : MGFWNRSNKTSD-------------------VDLQDITTTTDHEKNPHPED---GHPAVSDD----ALP---AEDVTEGVKDMEAITLVWSKA-S-----L
S.pomb 1 : MEAKETHSISDHE--------VELQDA-KPEEKSENGNFVFEKAFSSDEEK---GSGYNTNET----YSKMDNSLQHRGVSKIEAVRDSIYQN-KRGMYLA
C.neof 1 : MSRPSVDAPSVVS---AINQRPAFERVATDHKDAKPDPTDLEINHLHQDDD---K---SSIEIEQ-----------SAGVTKIEALYLVFGNG-WK----L
P.bras 1 : MVRQTTSVRSYGTCESAPHMEDRGEDAGLLYIEPRATSTDCYDDENDTNANIDGRDPT---RGSGYS--SASSDSMQEGVRKIEAISKTWTKK-S-----L
M gv E v l

S.sche 73 : WLLGISIFLTMFVYALDQGITSTIFSTLAASTFGVHSQIGTVSTASQIIRAISKPFIGKIADITSRPTTYVIILVFYVVGFAVAASASNFASYTVGVCFTS
G.zeae 70 : WLLGVSIGLTMFAYALDMGITTTIFGTLAASTFGVHSQLGTVNTAGQIIRAISKPFIGKLADITSRPTTYVVVLVFYAVGFAVAASASGFTSYVIGICFTS
C.glob 77 : WLLGISIGLTMFAYALDQSITSSIFTTMASSTFGQHSSLAAVSTASQIIRAISKPFIGKLADITSRPTTYVVILVFYVVGFAVAASASTFAAYTVGICFTS
P.nodo 73 : FILAASIGLTMFGYALDQGITSQ-FTVIAASAFSHHAEIGAVNTASSIIRAISKPFIGKLSDITSRPTTYVVVLVFYVIGFVIAATCKSIGAYIIGISFTA
A.oryz 90 : VTIIISLGLTMFVYAMDEGVTQQ-FTMIAASSFHMHAQLGAVNTASTVINGISKPVIGKLADVLSRPTSYIISLLFYVVGYAVAASCTNFVAYTVGVALTA
A.flav 60 : VTIITSLGLTMFVYAMDEGVTQQ-FTMIAASSFQMHAQLGAVNTASTVINGISKPVIGKLADVLSRPTSYIISLLFYVVGYAVAASCTNFVAYTVGVALTA
G.moni 67 : ICLFIFIWLVYLLNAFQSATVGN-LLPYVTSDWSAHSLLNTIGVVASSMTAAVFIPLAKLLDLWGRAEGYLLMVGFCELGLILMATSKDLSTYCAANVFYS
S.pomb 85 : YAFGIAILACSWASAIQSSTTYS-YQVYATASFNRTSMISTLEIATAIISSVCKPILGKFSDITSRMTYTLVLLFYVIGFIVVASSSTISAYVIGSVFIS
C.neof 77 : WTLWGSIALISIAYGLSQMTTYY-YTAFATSAFGEHTVLGTISVITGIMAGVAKPFLAKLADLFSRPWALALSVLFYTIGYIVVAASKSVADVAGGEVIYT
P.bras 91 : IVAYTGIFLMAFSTSL-EGQTVLSLSAYATSSFSKHSLISTVMVVQNVVNAVVKPMAKVADVFGRFEAFCIAVSIYVLGYVQMAASTDVQTYASAQIFYS
i l a t a s f h kp K D sRp y fy G A y g
MOTIF D1
MOTIF A
MOTIF B

S.sche 174 : IGKSGLDLLSDIIVADLTPLEWRGFFSACLSLPFIVTVPVNGFISS---GFYGN-WRWGLGMFAIMVPVLLMPAILTLYTIQRRGKQAGMVAMADSKDIRT
G.zeae 171 : VGKSGLDLLSDIIVADLTPLEWRGFFSACLALPFIVTVPVNGFIAE---GFYED-WRWGLGMFAILVPVLLLPAIFTLYTMQRRGEKAGMVTMADSKDVRT
C.glob 178 : VGKSGLDLLSDIIVGDLTPLQWRGFFGAALSIPFIITVPVNGFIAE---AFVDN-WRWGLGMFAILVPVLLVPAIFTLYSMQRRGEKMGMVTMAASKRLRT
P.nodo 173 : FGKSGLDLLGDIIVADLTPLEWRAFWSSMLSAPFIITVFINGFIAD---AMIPDHWRWGLGMFAIMMPVLLIPAIFTLYGVQRRADKLGAISFGEA--G-M
A.oryz 190 : VGKAGLNILCQIIVGDLTTLQWRGFWTSMIIAPYLVTTFTNGFVVD---AFVPDEWRWGLGMFAIMVPVLLTPAIIALYGTQQRARRMGIMGSS----S-A
A.flav 160 : VGKAGLNILCQIIVGDLTTLQWRGFWTSMIIAPYLVTTFTNGFVVD---AFVPDEWRWGLGMFAIMVPVLLTPAIIALYGTQQRARRMGIMGSS----L-A
G.moni 167 : VGFTGLIYSVDVMTADATNLKNRALAYAFTSSPYMISAFAGSYASDRML--ADIGWPWGFGTFAFITPVVCAPLYLLLKINLRKAKK-NIL-P--------
S.pomb 185 : IGSSGLDYLNTLVVGDLTSLKWRGFMTALLSTPYIATVWFTGFIVQ---GIIDSNWRWGYGMFAIIMPAVMTPAVIILMYLERQANKDENIKKII-----N
C.neof 177 : IGNTGLDFITSILLADITSLQWRGLVIGLYSLPFIPFAFVAGNIADGINAYSANGWRWGYGMFCIMIPCVVIPAILVLFWADWRAKKIGALSLASSTYARE
P.bras 191 : AGSTGLQILQQVFIADTSSLLNRALFSSLPDLPFLVTVWIGPMIAAAIL--RETSWRWGYGIWTIILPVAFLPLGLALFVNQRKAKQLNLLKP--------
G GL l D t L wR P t g WrWG Gmfai Pv Pa L
MOTIF C
MOTIF H

```
S.sche 271 : GRT-EASTG------SIAYWAHLAYQGLIDIDIFGLFLLGFAFSLILLLPITLAGDAKNGWHNPSMIAMIVVGFVFLILFALFE-YF-VARKPLMTRNILNN
G.zeae 268 : GRT-EATPK------NLTYWAKLAYRGLIDIDIVGLILLGFAFSLILLLPITLAKSAKGGWNNPSMVAMIVVGFVVLILFGLYE-YF-LAPKPMMTKRILQN
C.glob 275 : GDGVVASEEPTTNSTGAAYWMKLLYQGLVDIDIIGLVILGTSFSLILLLPFTLAKEADGGWANGSVIAMLVVGFVLLGAFVLFE-VY-VAAKPLMTKRILQN
P.nodo 268 : ARREGIKVR------TGKDYLNLAYRGIIDIDLAGLILLGVGFSLVLLSFNLTKTANGGWSNPSMIAMIVTGFVVLGFFAAFE-IF-FAPKPIMTRRIFRN
A.oryz 283 : --------EK------GEQTTLYTAWQCLVAIDIPGLVLLGFSFSLILLLPLSLAESAENGWNNRSMIAMEATGFAILVLFVVFE-IY-LAPKPMMTKRIIAN
A.flav 253 : --------EK------GEQTTLYTAWQCLVAIDIPGLVLLGFSFSLILLLPLSLAESAENGWNNRSMIAMEATGFAILVLFVVFE-IY-LAPKPMMTKRIIAN
G.moni 256 : -------KKA------SGRTLKESIWHYVLEFDVLGVFLFAAGLIIFLLPFTIASMAPHGWSTGYIIAMIIVGFILLVGFALNE-VY-FAPVPFLKFHFLTD
S.pomb 278 : YQTEEKNKN------KQSKWQ-KLWKAVLEVDLFGLILLGVGWSILLLLPFSLTSYAKNGWKNPSMIAMMVVGGVILIAYSGYE-MF-IAPYPSCPRRVM-N
C.neof 278 : KLLAGQTVE------KRPFIA-TCLYYARRIDAVGLLLMGFAFGCILSPFTLYTTAKGGYKNPSLIALLVVGGVLFISFCLWE-WK-VASHPIMPKRVF-N
P.bras 282 : -------KPW------KGRSFTSIVRKTWYDIDVFGLLLLSAGLALILIPVTLAANAKNKWKNNSIIAMVVVGGVCLLVFPLWESLKRVAPHPLLSLHLLKQ
                                                  D Gl llg      Llp l  A gw n s iAm  G  l f  E    A P        n
                                                  MOTIF E

S.sche 363 : RAFIAGVIIHTFNQLASAVR-NTYFSSYILNIKQWTTYQWTIFLGITTMGLCIVGPCVGLIHRTTHRYKTVMVLGAAAKVLGYGLLIQGNGNMTQDTVRLV
G.zeae 360 : RAFIAGVIIHIFNQMASSVR-NTYFSSYILIIKEWTTYQWTIFLGITTMGLCLVGPIVGLIHRVSHRYKGLMIFGAAARILGYGLLISPNGMMTEDTARLV
C.glob 374 : RTFLASVTIYTFNQMASATR-NTYLSSYVYIIKEWTVYEWTIFLGITTMGLSIMGPIVGLIQRSTHRYKSMMVFGAAARLISYGILVQSNGNMVQDTARLI
P.nodo 361 : RAFICALLVNVFNQMASATR-NNYFSSYVYIIKEWSNYSWNTFLNTTTLVLCIFGLLGGLIHRYSHRYKSLMVLGAVLKLIGTCIQMTSDLRSTQSTAALV
A.oryz 369 : KVFLAALGANLFDQMTTTLG-SNYFSSYIYIIKGWNNYTWTVFTGARNLAITIFSLVGGFLQVRYHRYKTQMIIGAVLKVVGYATCFTSNQRSTQSTAALA
A.flav 339 : KVFLAALGANLFDQMTTTLG-SNYFSSYIYIIKGWDNYTWTVFTGARNLAITIFSLVGGFLQVRYHRYKTQMIIGAVLKVVGYATCFTSNQRSTQSTAALA
G.moni 343 : RTLVGACLLDLTYQISYYCW-NNYFTSFLQVVNYLTVAEAGYVNNTFNVVSGFLLFLVGWGIRKTGYFKWLLWAGVPLYILAQGLMIYF-RNPTGYVGYLV
S.pomb 369 : RTFITAVIIDFFYYLAGYLQ-SMYFTTYTWILYDWSYRDWTYFNNTMTIALCVFGVFAGAMHRVFHRYKYLQIIGLVIKIVGYGILIRPNFAATGKVD-LA
C.neof 369 : RTFVCCLLIDWNYYLSGYLS-DTYWSSWLYVVKDYNDKDYTYIMNILTVGLCFFSVLAGLVQRYTHRFKYLQLSGLAIRIIGMGLNYLSVNGNMSD-GVIV
P.bras 371 : RTALAGCALAFFYFMAFYSSVQPYLYSYLQVVQDQSVTTAGRVTQTFSFTSTIAAVSISFVIRATRRYRIFVTSGCCIYIFGLFLMLFCNKPGASVPSTFA
             r f        f          Y sy                                 g  r  hryk    G   g                l

S.sche 463 : AAQLIFCLSS-LNVVGARVSVQASVPHKDIASLISIITLWSTLGSSVGSAVASAIWTNQMLDQMRVELPG-VPESTIKTVYGSIRSLR-KYDFNDPVRQGS
G.zeae 460 : AAQLIFCLSS-LNVVGVRVGVQASVPHDDIASLISIITLWSTLGSSVGSAIATSIWTEQMVDQMHVELPD-VDDTTIATIYGNIKTLK-KYDFFDPIRQGS
C.glob 474 : VAQLVFCLGS-FNVVGARVGSQASVPHEDMASIIALLTLWSTLGSSIGSAVSSAIWTSEMLDRMHLEMPG-VDEATIAKLYGNIKTLRTAYDFADPVRQGA
P.nodo 461 : MSQLLLGCGA-FTVIGARVGAQASVPHEDLSSAIAITSLWSTLASSVGYTIAASIWTEHMLPYMREEMPS-VPEKTIQTIYGSIKTLRTKYDWQDPIRQSA
A.oryz 469 : ISQVLLGTSA-LTALGSRIGAMASVPHEDMASIIAAYFLWTYLGSAAGYAIASAIWTDKMLGFMRDELPD-TPDSTLKKIYGSVDILRTQYDLDDPIREGA
A.flav 439 : ISQVLLGTSA-LTALGSRIGAMASVPHEDMASIIAAYFLWTYLGSAAGYAIASAIWTDKMLGFMRDELPD-TPDSTLKKIYGSVDILRTQYDLDDPIREGA
G.moni 442 : MCQIFISVGGSVFTICMQLAVLAAVDHQHVAAALAMLSVTGTAGDSIGDTISGAIWTNTFEKALKMYLPA-SALENLDAIYGDLPTQL-SYAKGTPERIAI
S.pomb 468 : WSLILIGMGGSFSVVGSQVSCQASVPHQDLAIASSLLPLYTNIGGAIGAAIASPIFSNKVPKYLREYLPSSINDTQVYNFYSDSSLIR-EYPVGTEIRDGA
C.neof 468 : MSRVLISIGGGISVTSSQVACQGSVAHQDMALAMAILALWTSIGGAIGSAISASVWNRRVPEALTKYLGSTHNSTEIAEIFGSIVIARTT----EP-RDLV
P.bras 472 : I-QTIIGLGAGFLNVPVQLGVQASAKHQEVAAATAMFLTCLEMGGAVGSAISGAVWTSSIPKKLALYLPE-ESRGDAEEIFGKLTKAL-SYPLGSATRIAI
               q                    asv H d a        l  g   G ai  iwt          lp          yg        y    p R
                                                         MOTIF F
```

```
S.sche 561 : IRAYAIVNGHITTASICLSVVTLFASVCMPNFYLGKQQNAVDNKGL--------DGSSIDVPQNRKEDT-STTTR-P--FWKKLVT-----LYYK
G.zeae 558 : IRAYSIVNGHITIVSIVFACIPLIASFFMPNFYLGKQQNAVNNKGL--------DGEVVDVPKNGATDVNSTETK-PATFMQKMAA-----LYRK
C.glob 573 : IRAYAYVNGHIATTALVMAAVPLIATFFMPDFYLGNQQNAVTNLGL--------DGERVDVPERASEDQMGAKKT-SKPFYRRLID-----GYYK
P.nodo 560 : VRAYTRVNGIIFIAAIVLNVVPVILSICMPDYYLGKQQNAVTNTGV--------DGQPVQVQGRRNETKKPGIFAKVKRFYR----------RET
A.oryz 568 : IRAYTRTNGIIFIVAAAISTLSILCSFLMPDYYLGKQQNAVTNLGL--------DGKPVDIPTCGKNERS-FWTRIKYIISR----------RRS
A.flav 538 : IRAYTRTNGIIFIVAAAISTLSVLCSFLMPGNSLFLFPLFTTGFML--------T----------------------FLL--------------S
G.moni 541 : QKAYGYAQTRMLAAGTAIMALSFIWVALIRNLKVSEMRQTK-------------------------------------------------GNVF
S.pomb 568 : IKAYSRSMFFLLVPAVSLSFIPLAAAFWQSNFYLGNQQNAVEGDQD--------HKKKGD--------K-ET--T--------QEE-----KIII
C.neof 564 : ITAYNEAIKPLYLAALIMSMLSLAFGIFTYDIHLGKNHNAIEKRETAIRSEDEVRPEVIAAKAREVEEKIAAEVL-RKEQLQAQVERTNVKAGEE
P.bras 570 : NRAYDETFHRLLVIALCMAIPIIPLSLLMTNYRLDKMDTRPKVESIGDGEGEG-------LISRPSHDESPDRSS--------RFR-----RHFA
                AY                             l
```
