# Supplementary material for: Interaction of the heterotrimeric G protein alpha subunit SSG-1 of Sporothrix schenckii with proteins related to stress response and fungal pathogenicity using a yeast two-hybrid assay
Source: BMC Microbiol. 2010 Dec 9;10:317. doi: 10.1186/1471-2180-10-317 (PMC3018405; doi:10.1186/1471-2180-10-317)
Supplement: Additional file 5 — Protein multiple sequence alignment of SsGAPDH to other fungal GAPDH homologues. Multiple sequence alignment of the predicted amino acid sequence of S. schenckii SsGAPDH and GAPDH homologues from various fungi. In the alignment, black shading with white letters indicates 100% identity, gray shading with white letters indicates 75-99% identity, gray shading with black letters indicates 50-74% identity. [file 1471-2180-10-317-S5.PDF]

```
S.sche   1 : MV-VKAGINGFGRIGRIVFRNAIEHGDVEVVAVNDPFIDTNYAAYMLKYDSTHGAFKGEIKVEA-NGLNVNGKSVRFYQERDPAAIPWKDTGAEYVVEST
P.anse   1 : MT-VKVGINGFGRIGRIVFRNAVEHPDVEIVAVNDPFIEPKYAEYMLKYDSTHGVFKGTIQVSG-SDLIVNGKTVKFYTERDPSAIPWKDTGAEYIVEST
C.glob   1 : MA-IKVGINGFGRIGRIVFRNAVEHADVEIVAVNDPFIEPKYAEYMLKYDSTHGVFNGTIAVEG-SDLVVNGKKVKFYTERDPAAIPWKDTGAEYIVEST
B.bass   1 : MAPVKVGINGFGRIGRIVFRNAVEHNDIDVVAVNDPFIEVKYAAYMLKYDSSHGLFKGEVAIDG-NDLVINGKKVRFYGERDPAAIPWKETSAEYVVEST
G.zeae   1 : MAPIKVGINGFGRIGRIVFRNAVEHPDIEVVAVNDPFIEPHYAVYMLKYDSSHGIFKGEVGNQG-NDLVVNGKTIKFYSERDPANIKWSETGADYVVEST
M.gris   1 : -M-VKCGINGFGRIGRIVFRNAIEHPDCEIVAVNDPFIEPKYAKYMLEYDSTHGRFKGTVEVSG-SDLVVNGKKVKFYTERDPANIPWSETGAEYVVEST
P.bras   1 : MV-VKVGINGFGRIGRIVFRNAVEHDDVEIVAVNDPFIETNYAAYMLKYDSTHGQFKGDIEHSGGNNLTVNNKTIHFYQERDPTNIPWGKHGVDYVVEST
N.cras   1 : MV-VKVGINGFGRIGRIVFRNAIEHDDIHIVAVNDPFIEPKYAAYMLRYDTTHGNFKGTIEVDG-ADLVVNGKKVKFYTERDPAAIPWSETGADYIVEST
S.macr   1 : MV-VKVGINGFGRIGRIVFRNAIEHDDIQIVAVNDPFIEPKYAEYMLRYDSTHGNFKGTIAVEG-SDLVVNGKKVKFYTERDPSAIPWSETGADYIVEST
H.sapi   1 : MGKVKVGVNGFGRIGRLVTRAAFNSGKVDIVAINDPFIDLNYMVYMFQYDSTHGKFHGTVKAEN-GKLVINGNPITIFQERDPSKIKWGDAGAEYVVEST
             m  vKvGiNGFGRIGRiVfRnA eh d   VAvNDPFIe  Ya YMl YDstHG FkG     g   L vNgk   fy ERDP  IpW  tga Y VEST

S.sche  99 : GVFTTTDKAKAHLAGGAKKVIISAPSADAPMYVIGVNEKTYDGKADVISNASCTTNCLAPLAKVINDKFGIVEGLMTTVHSYTATQKTVDGPSAKDWRGG
P.anse  99 : GVFTTTEKASAHLKGGAKRVIISAPSADAPMYVMGVNEKTYDGKAAVISNASCTTNCLAPLAKVVNDKFGIVEGLMTTVHSYTATQKTVDGPSAKDWRGG
C.glob  99 : GVFTTTEKAGAHLKGGAKRVIISAPSADAPMYVMGVNEKTYDGSAQVISNASCTTNCLAPLAKVVNDKFGIVEGLMTTVHSYTATQKTVDGPSAKDWRGG
B.bass 100 : GVFTTTEKAKAHLQGGAKKVIISAPSADAPMYVMGVNEKSYDGSADVISNASCTTNCLAPLAKVIHDKFGIVEGLMTTIHSYTATQKTVDGPSAKDWRGG
G.zeae 100 : GVFTTIDKAKAHLQGGAKKVIISAPSADAPMYVVGVNENKYDGSADIISNASCTTNCLAPLAKVINDKFGIVEGLMTTVHSYTATQKTVDGPSSKDWRGG
M.gris  98 : GVFTTTDKASAHLKGGAKKVIISAPSADAPMYVMGVNEKSYDGSASVISNASCTTNCLAPLAKVINDKFGIVEGLMTTVHSYTATQKTVDGPSAKDWRGG
P.bras 100 : GVFTTTEKAKAHLSGGAKKVIISAPSADAPMFVMGVNEKSYRPDISVLSNASCTTNCLAPLAKVIHDNFGIAEGLMTTIHSYTATQKTVDGPSHKDWRGG
N.cras  99 : GVFTTTEKASAHLKGGAKKVIISAPSADAPMYVMGVNNETYDGSADVISNASCTTNCLAPLAKVIHDNFTIVEGLMTTVHSYTATQKTVDGPSAKDWRGG
S.macr  99 : GVFTTTEKASAHLKGGAKKVIISAPSADAPMYVMGVNNESYDGSANVISNASCTTNCLAPLAKVIHDNFTIVEGLMTTVHSYTATQKTVDGPSSKDWRGG
H.sapi 100 : GVFTTMEKAGAHLQGGAKRVIISAPSADAPMFVMGVNHEKYDNSLKIISNASCTTNCLAPLAKVIHDNFGIVEGLMTTVHAITATQKTVDGPSGKLWRDG
             GVFTTt KA AHL GGAK VIISAPSADAPMyVmGVN   Ydg a viSNASCTTNCLAPLAKVi D FgIvEGLMTTvHsyTATQKTVDGPS KdWRgG

S.sche 199 : RGAAQNIIPSSTGAAKAVGKVIPELNGKLTGMSLRVPTANVSVVDLTARLEKGASYDEIKAAIKEASEGPLKGIFGYTEDDVVSSDLNGNLNSSILDAKA
P.anse 199 : RGAAQNIIPSSTGAAKAVGKVIPELNGKLTGMAFRVPTSNVSVVDLTCRLEKPASYETIKAALKEASEGELKGILGYTEDEIVSSDLNGNANSSIFDAKA
C.glob 199 : RGAAQNIIPSSTGAAKAVGKVIPELNGKLTGMSFRVPTSNVSVVDLTCRLQKEASYDEIKAALKEASEGDLKGILGYTEDEIVSSDLNGNPNSSIFDAKA
B.bass 200 : RGAAQNIIPSSTGAAKAVGKVIPELNGKLTGMSMRVPTANVSVVDLTARLEKGASYDAIKAAIKEAADGPLKGILGYTEDEVVSTDLNGNTNSSIFDAKA
G.zeae 200 : RGAAQNIIPSSTGAAKAVGKVIPELNGKLTGMSMRVPTANVSVVDLTVRLEKGASYDQIKQVIKEASEGDLKGVLAYTEDDVVSSDLNGNTNSSIFDAKA
M.gris 198 : RGAAQNIIPSSTGAAKAVGKVIPALNGKLTGMSMRVPTANVSVVDLTCRLEKGASYEEIKAAIKEAADGPLKGILEYTEDDVVSSDMIGNNASSIFDAQA
P.bras 200 : RTAAQNIIPSSTGAAKAVGKVIPALNGKLTGMAMRVPTANVSVVDLTCRTEKPVTYDQIKAAVKAASEGELKGILGYSEDALVSTDLNGDPRSSIFDASA
N.cras 199 : RTAAQNIIPSSTGAAKAVGKVIPDLNGKLTGMAMRVPTANVSVVDLTARIEKGATYDEIKEVIKKASEGPLAGILAYTEDEVVSSDMNGNPASSIFDAKA
S.macr 199 : RTAAQNIIPSSTGAAKAVGKVIPDLNGKLTGMSMRVPTANVSVVDLTVRIEKGASYDEIKEVVKKASEGPLAGILAYTEDEVVSSDMNGNPASSIFDAKA
H.sapi 200 : RGALQNIIPASTGAAKAVGKVIPELNGKLTGMAFRVPTANVSVVDLTCRLEKPAKYDDIKKVVKQASEGPLKGILGYTEHQVVSSDFNSDTHSSTFDAGA
             R AaQNIIPsSTGAAKAVGKVIP LNGKLTGM  RVPTaNVSVVDLT R eK a Yd IK   K AseG LkGil YtEd  VSsD ngn  SSifDA A

S.sche 299 : GISLNPNFVKLVSWYDNEWGYSRRVVDLISYVAKVDSS-H
P.anse 299 : GISLNDNFVKLVSWYDNEWGYSRRVLDLLSYVAKYDAS-H
C.glob 299 : GISLNKNFVKLVSWYDNEWGYSRRVLDLLAYVAKVDAS-K
B.bass 300 : GISLNDNFVKLVSWYDNEWGYSRRVLDLISYVAKVDAS-K
G.zeae 300 : GISLNDNFVKLVSWYDNEWGYSRRVLDLLAHVAKVDAS-K
M.gris 298 : GIALNDKFVKLVSWYDNEWGYSRRVIDLVTYISKVDGG-K
P.bras 300 : GIALNDRFVKLISWYDNEWGYSRRVLDLIAYIAKVDAG-K
N.cras 299 : GISLNKNFVKLVSWYDNEWGYSRRVLDLISYISKVDAKKA
S.macr 299 : GISLNKNFVKLVSWYDNEWGYSRRVLDLISYVAKVDASKA
H.sapi 300 : GIALNDHFVKLISWYDNEFGYSNRVVDLMAHMA---SK-E
             GI LN  FVKLvSWYDNEwGYSrRV DL  y akvd
```
